# Molecular and functional characterization of *Schistosoma japonicum* annexin A13

**DOI:** 10.1186/s13567-023-01244-z

**Published:** 2023-12-04

**Authors:** Haoran Zhong, Ling Hou, Fanglin Qin, Yuqi Ren, Bowen Dong, Danlin Zhu, Hao Li, Ke Lu, Zhiqiang Fu, Jinming Liu, Shaopeng Gu, Yamei Jin

**Affiliations:** 1 National Reference Laboratory for Animal Schistosomiasis, Shanghai Veterinary Research Institute, Chinese Academy of Agricultural Sciences, Shanghai, China; 2 Key Laboratory of Animal Parasitology of Ministry of Agriculture and Rural Affairs, Shanghai Veterinary Research Institute, Chinese Academy of Agricultural Sciences, Shanghai, China; 3 College of Animal Science and Veterinary Medicine, Shanxi Agricultural University, Shanxi, China; 4 College of Life Sciences, Shanghai Normal University, Shanghai, China

**Keywords:** *Schistosoma japonicum*, annexin A13, development, immune protection, host–parasite interaction

## Abstract

Schistosomiasis is a neglected tropical disease that affects humans and animals in tropical and subtropical regions worldwide. Schistosome eggs are responsible for the pathogenesis and transmission of schistosomiasis, thus reducing egg production is vital for prevention and control of schistosomiasis. However, the mechanisms underlying schistosome reproduction remain unclear. Annexin proteins (ANXs) are involved in the physiological and pathological functions of schistosomes, but the specific regulatory mechanisms and roles of ANX A13 in the development of *Schistosoma japonicum* and host–parasite interactions remain poorly understood. Therefore, in this study, the expression profiles of SjANX A13 at different life cycle stages of *S*. *japonicum* were assessed using quantitative PCR. In addition, the expression profiles of the homolog in *S. mansoni* were analyzed in reference to public datasets. The results of RNA interference showed that knockdown of SjANX A13 significantly affected the development and egg production of female worms in vivo. The results of an immune protection assay showed that recombinant SjANX A13 increased production of immunoglobulin G-specific antibodies. Finally, co-culture of *S. japonicum* exosomes with LX-2 cells using a transwell system demonstrated that SjANX A13 is involved in host–parasite interactions via exosomes. Collectively, these results will help to clarify the roles of SjANX A13 in the development of *S. japonicum* and host–parasite interactions as a potential vaccine candidate.

**Supplementary Information:**

The online version contains supplementary material available at 10.1186/s13567-023-01244-z.

## Introduction

Schistosomiasis is a zoonotic parasitic disease that affects approximately 290 million people globally and is responsible for 1.4–3.3 million disability-adjusted life years annually [1, 2]. Among the three main pathogenic species of schistosomes, *Schistosoma japonicum* infects more than 40 kinds of animals, resulting in significant economic losses to the livestock industry [3]. Livestock is recognized as a key contributor to animal schistosomiasis in Asia [4]. Due to the large amount of feces produced by livestock and high degree of environmental overlap between humans and livestock during agricultural production, schistosome eggs released by infected animals threaten human health [5]. The increasing attention to animal schistosomiasis over the past few decades in China has facilitated significant improvements in agricultural practices [6]. National surveillance of schistosomiasis-related morbidity in China has detected no case of *S. japonicum* infection of livestock from 2017 to 2022 [7–10]. However, vast areas of China are suitable habitats for snails, which serve as intermediate hosts for schistosomes. Southeast Asian countries neighboring China, as well as some developing countries in Africa, are still suffering from severe schistosomiasis [11, 12]. In addition, the World Health Organization recommends praziquantel (PZQ) for treatment of schistosomiasis, which has led to the emergence of drug-resistant phenotypes, thereby further complicating prevention and control of schistosomiasis [13, 14]. Therefore, the development of vaccines or to explore novel drug targets against schistosomiasis is still necessary on a global basis.

Unlike most trematodes, which are hermaphroditic, schistosomes are dioecious, presenting as separate male and female sexes [15]. The sexual development and maintenance of the mature reproductive status of female worms require perpetual coupling with males [16]. Maturation and egg production lead to the pathogenesis and transmission of schistosomiasis. Hence, elucidation of the molecular mechanisms of the pathogenesis and transmission is vital for prevention and control of schistosomiasis. A proteomic study conducted by our group identified several highly expressed proteins involved in reproductive development of mated female (MF) worms and single-sex infected female (SF) worms at 18, 21, 23, and 25 days (four important time points from sexual maturation to egg production) and various recombinant proteins with immunoprotective effects [17, 18]. Subsequent analysis of *S. japonicum* identified several highly expressed proteins, including annexin A13 (SjANX A13), in MF worms at all four time points.

Multifunctional ANX proteins (ANXs) are expressed by a wide range of parasites [19–21]. Recent omics studies have identified several ANXs in the gut, vitelline gland, and tegument of *S. mansoni* [22–24], *S. japonicum* [25] and *S. bovis* [26]. Tissue-specific transcriptomic analyses of female *S. japonicum* found that SjANX B7 and B22 were expressed in the gut lining, while SjANX B5 was expressed in the vitelline gland, suggesting potential developmental roles in females [27]. Transcriptional profiling revealed that expression of SmANX B22 and B30 during schistosome development was correlated with life cycle stages characterized by the presence of the syncytial tegument layer [24, 28–30]. Besides, the native ANX protein of *S*. *bovis* (SbANX) was expressed on the tegument surface of the schistosomula and adult [26]. ANXs are frequently identified in the tegument and present important antigen sources for anti-schistosome vaccines and promising targets for drug development [31]. Although many ANXs have been identified in schistosomes, SjANX A13 is a promising vaccine candidate due to roles in the growth and development of *S. japonicum.*

In addition, schistosome ANXs are components of excretory/secretory products [32] and exosomes [33], which may mediate the secretion of anticoagulant ANXs and restrict host hemostatic responses to facilitate blood feeding. Thus, the identification and characterization of ANXs released from parasite excretory/secretory products or exosomes and crucial interactions with host cells could help to clarify host–parasite interactions.

Here, the molecular features of SjANX A13 and transcriptional levels at different maturation stages were assessed. RNA interference (RNAi) was conducted to explore the functional roles of SjANX A13 in growth, development, and fecundity of *S. japonicum*. In addition, the protective immunity of recombinant SjANX A13 (rSjANX A13) was assessed in a mouse model. Finally, the potential involvement of SjANX A13 in host–parasite interactions through exosomes was investigated.

## Materials and methods

### Parasites and animals

Specific-pathogen-free (SPF) male BALB/c mice (6–7 weeks old; body weight 18 ± 2 g) were purchased from Shanghai Jiesijie Laboratory Animal Co., Ltd. (Shanghai, China) and housed in SPF-grade animal rooms at the Shanghai Veterinary Research Institute, Chinese Academy of Agricultural Sciences (Shanghai, China).

The Chinese strain of *S. japonicum* cercariae used in this study was obtained from the National Reference Laboratory for Animal Schistosomiasis, Shanghai Veterinary Research Institute, Chinese Academy of Agricultural Sciences (Shanghai, China). BALB/c mice were percutaneously infected by applying cercariae to the shaved skin of the abdomen. Depending on different experiments, animals were euthanized and worms were collected at specific time points through hepatic-portal perfusion as previously described [34].

### Sequence analysis of SjANX A13

Based on the amino acid sequence of SjANX A13 (GenBank no: CAX70812.1), the cDNA sequence of this protein was found in the *S. japonicum* genome sequence. Protein domains were analyzed by the Simple Modular Architecture Research Tool (SMART) [35]. The physicochemical properties (molecular weight, isoelectric point, transmembrane region, signal peptide) of SjANX A13 were analyzed using the Detaibio tool [36]. Alignments of multiple ANXs with the highest homology in other species included *Schistosoma mansoni* (XP_018652854.1), *Schistosoma haematobium* (XP_035587885.1), *Homo sapiens* (AAI25159.1) and *Mus musculus* (NP_081487.1) were performed using the ClustalX 1.83 program [37].

### Phylogenetic analysis of SjANX A13

ANX A13 protein sequences of multiple species were acquired from the database of National Center for Biotechnology Information (NCBI). Amino acid sequences of ANX with the highest homology in other species including *Schistosoma* like *Schistosoma bovis* (CAH8605973.1), *Schistosoma guineensis* (CAH8598563.1), *Schistosoma intercalatum* (CAH8585262.1), *Schistosoma mattheei* (CAH8576064.1), *Schistosoma margrebowiei* (CAH8603292.1), *Schistosoma rodhaini* (CAH8645073.1) and *Schistosoma spindale* (CAI2731872.1); other trematodes (except *Schistosoma*) like *Fasciola.hepatica* (THD28190.1), *Opisthorchis felineus* (TGZ72241.1) and *Clonorchis sinensis* (GAA48684.1); cestoda like *Echinococcus granulosus* (KAH9278638.1) and *Taenia solium* (AAY17503.1); nematodes like *Caenorhabditis elegans* (CAE45742.1); mammals like *Homo. sapiens* (AAI25159.1) *Mus. musculus* (NP_081487.1) and *R.norvegicus* (NP_001128382.1) were used to construct the phylogenetic tree by MEGA-X software with the maximum likelihood method, then improved by the iTOL online tool [38].

### Molecular modelling and evaluation of SjANX A13

The 3-dimensional (3D) structure for SjANX A13 was modelled by homology modelling method using the SWISS-MODEL [39]. The protein modelling was performed based on the reference sequence of crystal structure of calcium-bound annexin (Sm)1 as a template (Uniprot accession number: C4QH88) [40]. The validation of the 3D model was assessed using Ramachandran plots [41], QMEANDisCo global and GMQE estimate [42].

### Bioinformatics analysis of SjANX A13

To get a general overview of the SjANX A13 expression in publicly available datasets, a volcano plot was presented to illustrate the SjANX A13 expression level in our previous proteomics data focused on 18, 21, 23 and 25-day SF and MF (dataset identifier PXD030834) [17]. In addition, the expression data of SmANX (GenBank no: XP_018652854.1, also referred as Smp_074140 obtained from WormBase ParaSite [43]) in a publicly RNAseq dataset [44] and a single-cell RNAseq (scRNAseq) dataset [45] of *S. mansoni* were taken as a reference for the expression of SjANX A13. A heatmap representing the Smp_074140 gene expression throughout life stage (egg, miracidium, sporocyst, cercaria, schistosomulum, 21-day juvenile, 28-day juvenile, 35-day adult, 38-day adult, adult older than 42-day) of mated male (MM), single-sex infected male (SM), MF and SF was constructed.

In addition, to explore the potential interacting molecules of SjANX A13, the online tool STRING v11.5 was used to construct a predicted interaction network [46]. Gene ontology (GO) [47] were performed to assess the molecular function, cellular component and biological process of the predicted interacting molecules, and Kyoto Encyclopedia of Genes (KEGG) [48] analysis were conducted to allocate proteins into metabolic and regulatory pathways.

### Expression examination of SjANX A13 in life cycle by qPCR

To collect SF from different life stages, the sexes of *S. japonicum* cercariae were first identified by PCR based on previous publication [49]. After determining the sexes of cercariae, mice were infected with unisexual cercariae percutaneously to obtain SF through hepatic-portal perfusion at different life stages. To collect MM and MF, mice were infected with bisexual cercariae and worms were collected and separated manually at each time point, except for 7-day and 14-day worms.

All worms were washed three times in phosphate buffer solution (PBS, Corning, USA) at 4 °C to remove residual host debris and total RNA was then extracted by TRIzol reagent (Invitrogen, USA) according to the manufacturer’s instructions [50]. The reverse-transcription was performed using a Hifair® III 1st Strand cDNA Synthesis SuperMix for qPCR kit (Yeasen, China) and the resulting cDNA was used as template for qPCR with Hieff® qPCR SYBR Green Master Mix (Yeasen, China). The relative mRNA expression levels of genes were quantified with β-tubulin served as an endogenous control. The LightCycler 96 system (Roche, China) was used for qPCR analysis. The cycling conditions were as follows: preincubation, 95 °C for 60 s; 2 step amplification, 95 °C for 5 s, and 60 °C for 30 s, for 40 cycles; melting, 95 °C for 10 s, 65 °C for 60 s, 97 °C for 1 s. All samples were assessed in triplicate. Subsequently, the relative transcript levels of SjANX A13 were analyzed using the 2^−ΔΔCt^ method [51]. The primers used in the qPCR analysis are listed in Table 1.

**Table 1:** **Primers for qPCR**

Gene name	Primer sequence (5′–3′)
SjAnnexin A13-RT-F	CTGGTGAAGCACGTCTAGGA
SjAnnexin A13-RT-R	CCAGATGTTTCCGATGCAAGTG
Sjtubulin A13-RT-F	ACCTCAACAACCACCACC
Sjtubulin A13-RT-R	TTGCGGCTTCTGCTCTTC
Human GAPDH-RT-F	GTCTCCTCTGACTTCAACAGCG
Human GAPDH-RT-R	ACCACCCTGTTGCTGTAGCCAA

### RNA interference (RNAi) assay in vivo

For biological characteristic analysis of SjANX A13, RNAi was performed using the method as described previously with modification [17, 52, 53]. Mice were exposed to approximately 200 cercariae and at 22 days post-infection (dpi), three specific siRNA (S1, S2 and S3) pairs and a non-specific (negative control, NC) siRNA pair, dissolved in 200 µL diethyl pyrocarbonate (DEPC)-treated water with the concentration of 100 nM, or 200 µL PBS were injected via the tail vein. After 48 h, worms were collected and the interference efficiency of these siRNA pairs was evaluated through qPCR.

After the interference efficiency was compared, SjANX A13 S1 was selected for long-term interference assay to assess the effect of SjANX A13 on the development and reproduction of *S. japonicum*. Mice were exposed to 40 cercariae, and SjANX A13 S1, NC siRNA (dissolved in 200 µL DEPC-treated water) or 200 µL PBS were given via the tail vein every 4 days since 4 dpi. Mice were sacrificed at 42 dpi, and worms were collected and counted. Part of worms were applied for interference efficiency assay with qPCR and the rests were prepared for morphological observation. In addition, livers were collected with the calculation of liver egg count and the liver egg-hatching rate according to previous studies [54, 55].

The siRNA pairs used in this study were synthesized by Shanghai GenePharma Co., Ltd. (Shanghai, China) and the detailed sequences of these siRNA pairs are shown in Table 2.

**Table 2:** **Sequences of SjAnnexin A13-specific siRNAs and the control siRNA**

Name	Sequence (5′–3′)	Target regions
SjAnnexin A13 S1 sense	GGUCCAAAUGGUGAAAUAUTT	72–91 bp
SjAnnexin A13 S1 anti-sense	AUAUUUCACCAUUUGGACCTT	
SjAnnexin A13 S2 sense	GGAAUAAGCGAUCCUAGAATT	486–505 bp
SjAnnexin A13 S2 anti-sense	UUCUAGGAUCGCUUAUUCCTT	
SjAnnexin A13 S3 sense	GCACAUUGAUGCGCAUUAUTT	943–962 bp
SjAnnexin A13 S3 anti-sense	AUAAUGCGCAUCAAUGUGCTT	
NC sense	UUCUCCGAACGUGUCACGUTT	Not applicable
NC anti-sense	ACGUGACACGUUCGGAGAATT	

### Morphological observation by scanning electron microscopy (SEM)

MM and MF worms from the long-term interference assay were washed with PBS for three times (15 min/time), and then fixed in 1% osmium tetroxide for 2 h, and then dehydrated in a graded ethanol series of 30%, 50%, 70%, 80%, 90%, 95%, and 100% for 20 min each. After desiccation in a CO_2_ critical point drying apparatus, the dehydrated worms were then coated with a gold film in a vacuum-coating device and then examined under a JSM-6380LV scanning electron microscope (JEOL, Ltd., Tokyo, Japan).

### Expression and purification of recombinant SjANX A13

Primers of SjANX A13 were designed based on its nucleotide sequence (Genbank no: FN315080.1) using the corresponding restriction enzyme sites of BamHI and XhoI at the N-terminus and C-terminus, respectively. The verified SjANX A13 cDNA fragment was amplified and ligated into the expression vector pET-28a(+) (Novagen, Germany). Products were transformed into *Escherichia coli* BL21 (DE3) cells (Invitrogen, USA), and recombinant clones were obtained by antibiotic selection. The recombinant proteins were overexpressed in the presence of isopropyl-b-d-thiogalactopyranoside (IPTG). Transformed cells were grown in Luria broth (LB) with 1 mg/mL kanamycin at 37 °C until OD_600_nm = 0.7, and IPTG was added to the culture at a final concentration of 1 mM. After 8 h induction, cells were harvested and the expression of recombinant protein was analyzed by SDS-PAGE. The histidine-tagged fusion recombinant protein was then purified from *E. coli* lysates by metal affinity chromatography using Ni-NTA His·Bind Resin Chromatography according to the manufacturer’s instructions (Novagen, China). The purified recombinant SjANX A13 (rSjANX A13) protein was analyzed by SDS-PAGE and the protein concentration was measured by a bicinchoninic acid (BCA) assay kit (Yeasen, China). The primers used in this study are listed in Table 3.

**Table 3:** **Primer sequences used to amplify SjAnnexin A13 gene**

Name	Primer sequence (5′–3′)	Underlined
SjAnnexin A13-F	CGCGGATCCATGGGAAGATCTAAACTTC	BamHI restriction enzyme sites
SjAnnexin A13-R	CCGCTCGAGTTAACTCCATTCAGCACC	XhoI restriction enzyme sites

### Vaccination, immune response assays and evaluation of immune protection

To explore whether SjANX A13 provides immune-protective effect, an immune protection trial was conducted. Mice were randomly distributed into three groups (ten mice each). Mice in the vaccination group were injected subcutaneously with rSjANX A13 emulsified with ISA206 adjuvant (Seppic, France) (50 µg/100 µL/mouse). Following this, the immunity of the mice was enhanced twice at intervals of 2 weeks with 25 µg rSjANX A13/mouse. Each mouse in the adjuvant and control groups was injected with 100 µL ISA206 adjuvant in PBS or 100 µL PBS only, respectively. Fourteen days after the last vaccination, all mice were challenged percutaneously with 40 *S. japonicum* cercariae and mice were sacrifice 42 days after challenge. Worms were collected and counted and blood samples were collected from mice in each group by retro-orbital bleeding [56]. Specific IgG antibodies against SjANX A13 were detected by ELISA in the serum, as described in a previous study [17].

### Cell line and cell culture

Human hepatic stellate cell (HSC) line LX-2 was obtained from Boster company (Wuhan, China) and cultured in the Dulbecco’s modified Eagle’s medium (DMEM, Corning, USA), supplemented with 10% heat-inactivated fetal bovine serum (FBS, Gibco, USA), 1% penicillin-streptomycin (Thermo Fisher Scientific, USA) in a humidified incubator at 37 °C with 5% CO_2_.

### Construction of transwell model

Mice were percutaneously infected with approximately 100 cercariae and worms were collected from the infected mice at 28 dpi. Worms were thoroughly and gently washed three times with 30 mL PBS and then maintained in preheated DMEM. In each transwell system of 12-well plate (PET membrane, pore size 0.4 μm) (Corning, USA), LX-2 cells (5 × 10^5^ cells/well) were placed in the lower well with 1.5 mL DMEM containing 10% FBS and 1% penicillin-streptomycin. Five pair adult worms were transferred from the previous DMEM culture and placed in the upper well with 900 µL fresh DMEM containing 10% FBS and 1% penicillin–streptomycin. A transwell insert with only an unused schistosomal medium was utilized as a control. Each transwell system had at least three technical replicates and were cultured in a humidified incubator at 37 °C with 5% CO_2_. Cells were harvested for qPCR analysis 48 h after co-incubation was established. The relative mRNA expression of SjANX A13 were quantified with GAPDH served as an endogenous control.

### Isolation and characterization of *S. japonicum* exosomes

The isolation of *S. japonicum* exosomes was performed as previous described with modification [33, 57]. Briefly, New Zealand rabbits were percutaneously infected with approximately 3000 cercariae and worms were collected through hepatic-portal perfusion at 28 dpi. Worms were gently washed 3–5 times with 50 mL PBS and the dead or fragmentary ones were discarded. The remaining worms were then maintained in preheated RPMI-1640 culture medium (Corning, USA) containing 1% penicillin-streptomycin at 37 °C under 5% CO_2_ at a density of ~15 worm pairs/mL for 2 h. After 2 h incubation, the supernatant was collected and fresh culture medium was added for the next collection (the whole collecting procedure could last for 3–4 days until the worms were less active). The pellets in the collected supernatant were discarded by centrifugation at 2000 × *g* and 14 000 × *g* for 30 min each at 4 °C, respectively. Then, the supernatant was collected and dialyzed in PBS for 24 h at 4 °C followed by centrifugal ultrafiltration through a 3K Omega membrane (Pall, USA). The supernatant was then filtered using a 0.22 μm syringe filter (Pall, USA) and a total exosome isolation kit (Thermo Fisher Scientific, USA) was used according to the manufacturer’s instructions. The exosome pellet was resuspended in 200 µL of PBS and then stored at −80 ℃ until further analysis. Each time before proceeding with downstream experiments, *S. japonicum* exosomes were subjected to transmission electron microscopy (TEM) analysis to confirm their morphology, and the concentration of *S. japonicum* exosomes was measured using a BCA assay kit according to the manufacturer’s instructions.

### Exosomes uptake experiment

The isolated *S. japonicum* exosomes were labeled with PKH67 using a Green Fluorescent Labeling Kit (Sigma Aldrich, USA), and the procedures were performed according to the manufacturer’s protocol. Briefly, 10 µg *S. japonicum* exosomes were stained with PKH67 dye in 500 µL of Diluent C fluid for 5 min at room temperature. Next, 1 mL 1% bull serum albumin (BSA, Yeasen, China) was added to stop the labeling process. Then, the labeled exosomes were re-purified via ultracentrifugation at 100 000 × *g* with PBS rinsing for 90 min. As a control for non-specific labeling of cells, PBS was PKH67-stained, washed, and added to the cells as a parallel experiment. The whole procedure was conducted at 4 °C. Then, the PKH67-labeled exosomes were co-incubated with LX-2 for 2 h in a humidified incubator at 37 °C with 5% CO_2_. Afterward, the culture medium was discarded, and the cells were washed in PBS three times, fixed with 4% formaldehyde solution (Servicebio, China) for 15 min and washed twice more with PBS, nuclei were stained with 4′,6-diamidino-2-phenylindole (DAPI, Sigma Aldrich, USA) for 3 min. After washed in PBS three times to remove the remaining DAPI, the cells were observed using a fluorescence microscopy (Olympus, Japan). In addition, to detect the expression of SjANX A13 in LX-2 cells after co-incubated with *S. japonicum* exosomes, cells were harvested for qPCR analysis 48 h after co-incubation. The relative mRNA expression of SjANX A13 gene were quantified with GAPDH served as an endogenous control.

### Statistical analysis

Data were analyzed with SPSS 25.0 software (SPSS Inc., USA) and expressed as mean ± standard deviation (SD) of three independent biological replicates. Data were statistically analyzed with Student’s *t*-tests. A *P*-value of < 0.05 was considered statistically significant in statistical analysis.

## Results

### In silico characterization of SjANX A13

**Figure 1:**
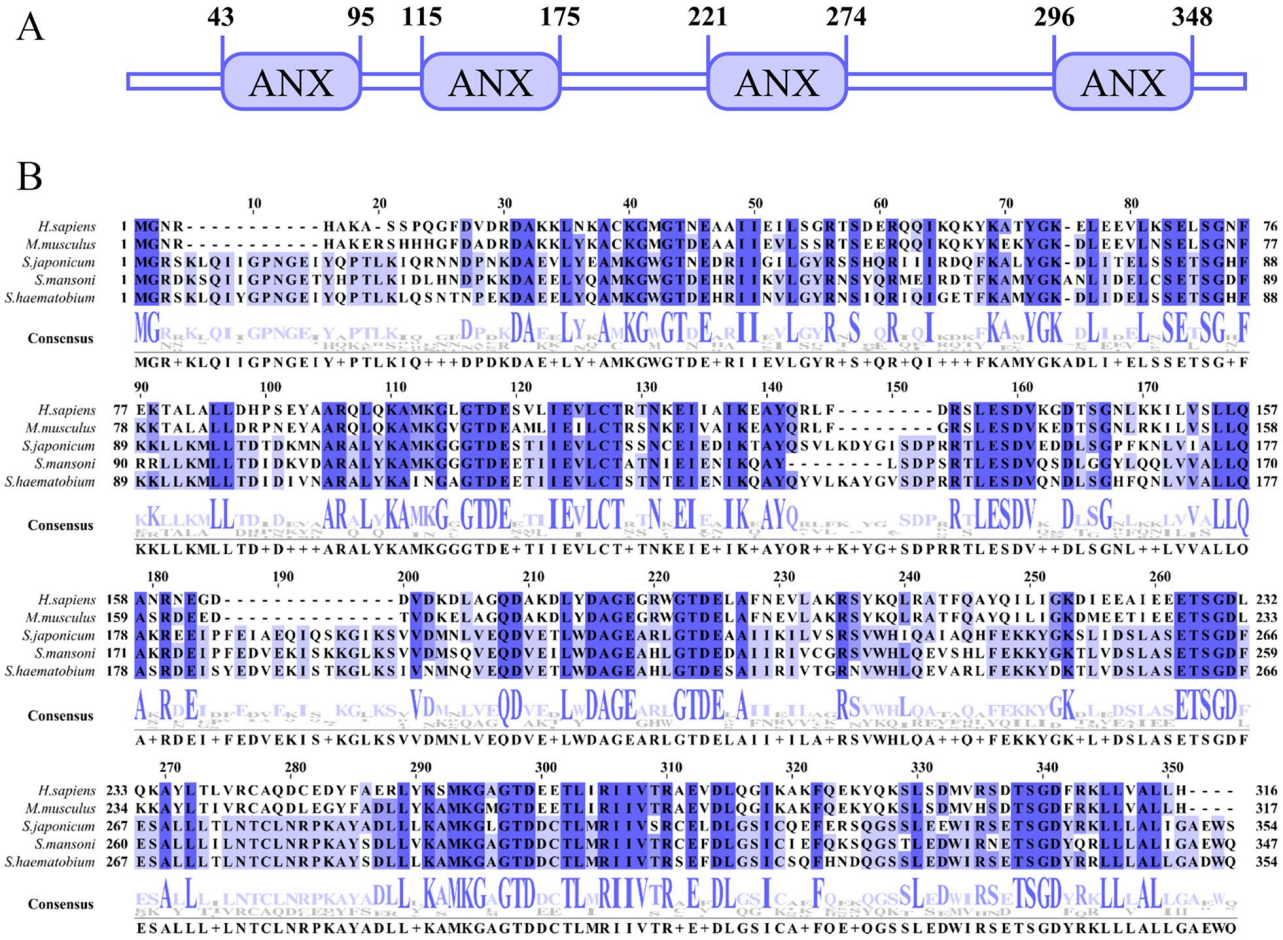
**Domain and multiple sequences alignment of SjANX A13. A** Protein domains of SjANX A13. **B** Comparison of amino acid sequences of SjANX A13 with associated sequences from other species, including *Schistosoma mansoni* (XP_018652854.1), *Schistosoma haematobium* (XP_035587885.1), *Homo sapiens* (AAI25159.1) and *Mus musculus* (NP_081487.1).

SjANX A13 (molecular weight, 39.62 kDa; isoelectric point, 5.11) consisted of 1253 bp and encoded 354 amino acid residues. Analysis with the SMART, which is used for the identification and annotation of genetically mobile domains and analysis of domain architectures, showed that SjANX A13 has four conserved repeat domains, each consisting of approximately 60 amino acids containing a calcium (Ca^+^)-binding motif (amino acids 43–95, 115–175, 221–274, and 296–348) (Figure 1A). In addition, sequence analysis revealed that SjANX A13 had no signal peptide structure and the whole protein was expressed extracellularly, therefore lacking a transmembrane helix, as confirmed by a model to predict membrane protein topology based on a hidden Markov model (Additional file 1). Sequence alignment showed that SjANX A13 shared 74.01%, 77.05%, 43.96%, and 44.27% identity with orthologs in *S. mansoni*, *S. haematobium*, *H. sapiens*, and *M. musculus*, respectively (Figure 1B).

### Phylogenetic analysis of SjANX A13

**Figure 2:**
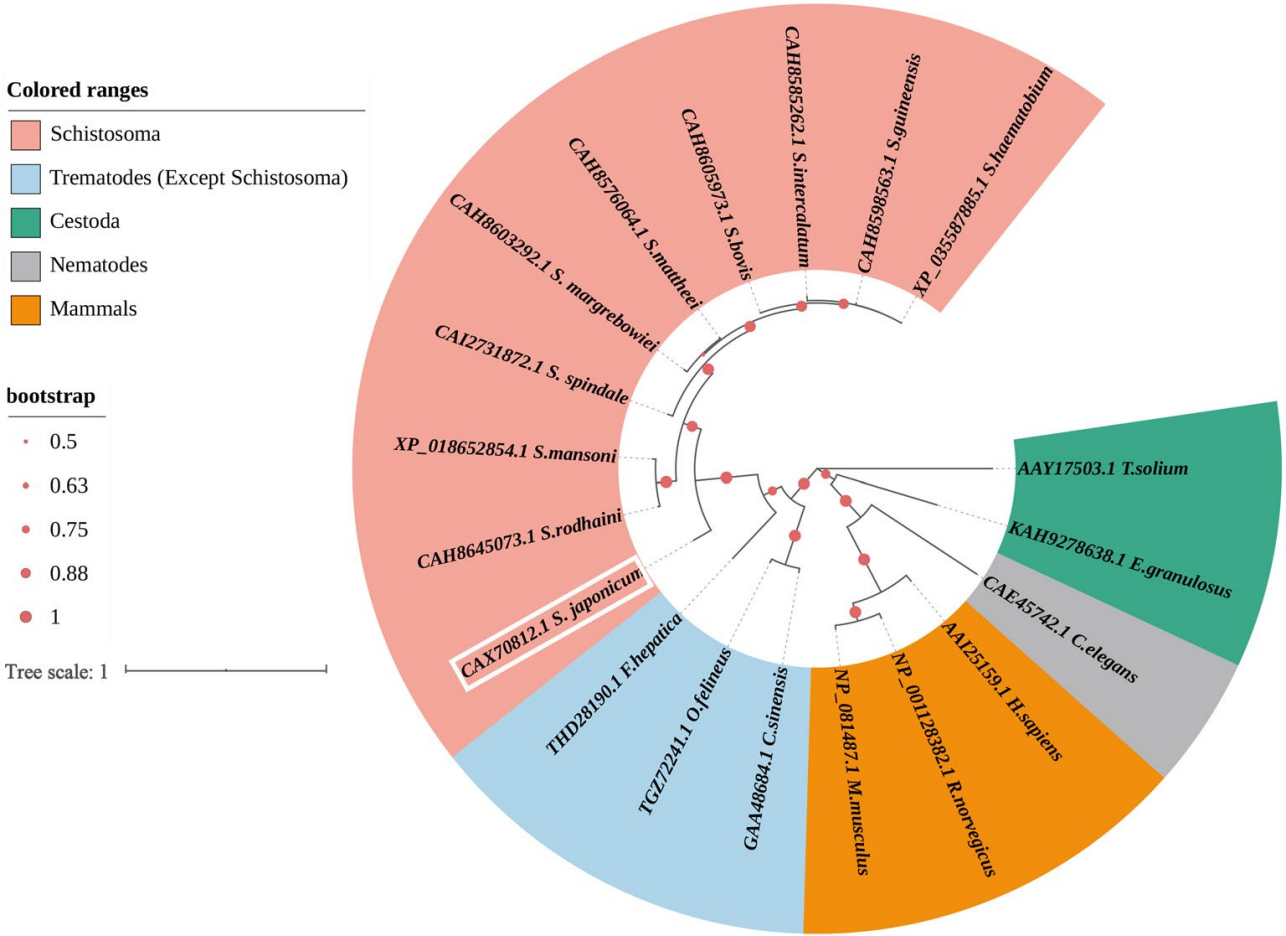
**Phylogenetic analysis of SjANX A13 and its homologues.** The sequence ID consists of the Genbank no.+ species name. Protein sequences were divided into five groups that are color-coded (see list to the top left of figure): *Schistosoma* like *Schistosoma bovis* (CAH8605973.1), *Schistosoma guineensis* (CAH8598563.1), *Schistosoma intercalatum* (CAH8585262.1), *Schistosoma mattheei* (CAH8576064.1), *Schistosoma margrebowiei* (CAH8603292.1), *Schistosoma rodhaini* (CAH8645073.1) and *Schistosoma spindale* (CAI2731872.1); other trematodes (except *Schistosoma*) like *Fasciola hepatica* (THD28190.1), *Opisthorchis felineus* (TGZ72241.1) and *Clonorchis sinensis* (GAA48684.1); cestoda like *Echinococcus granulosus* (KAH9278638.1) and *Taenia solium* (AAY17503.1); nematodes like *Caenorhabditis elegans* (CAE45742.1); mammals like *Homo. sapiens* (AAI25159.1) *Mus. musculus* (NP_081487.1) and *R.norvegicus* (NP_001128382.1).

ANX proteins of other species acquired from NCBI database were used to construct a phylogenetic tree, which showed that the *Schistosoma* genus has one clade and SjANX A13 is closely related to orthologs of all schistosome species (Figure 2).

### Molecular modelling and evaluation

**Figure 3:**
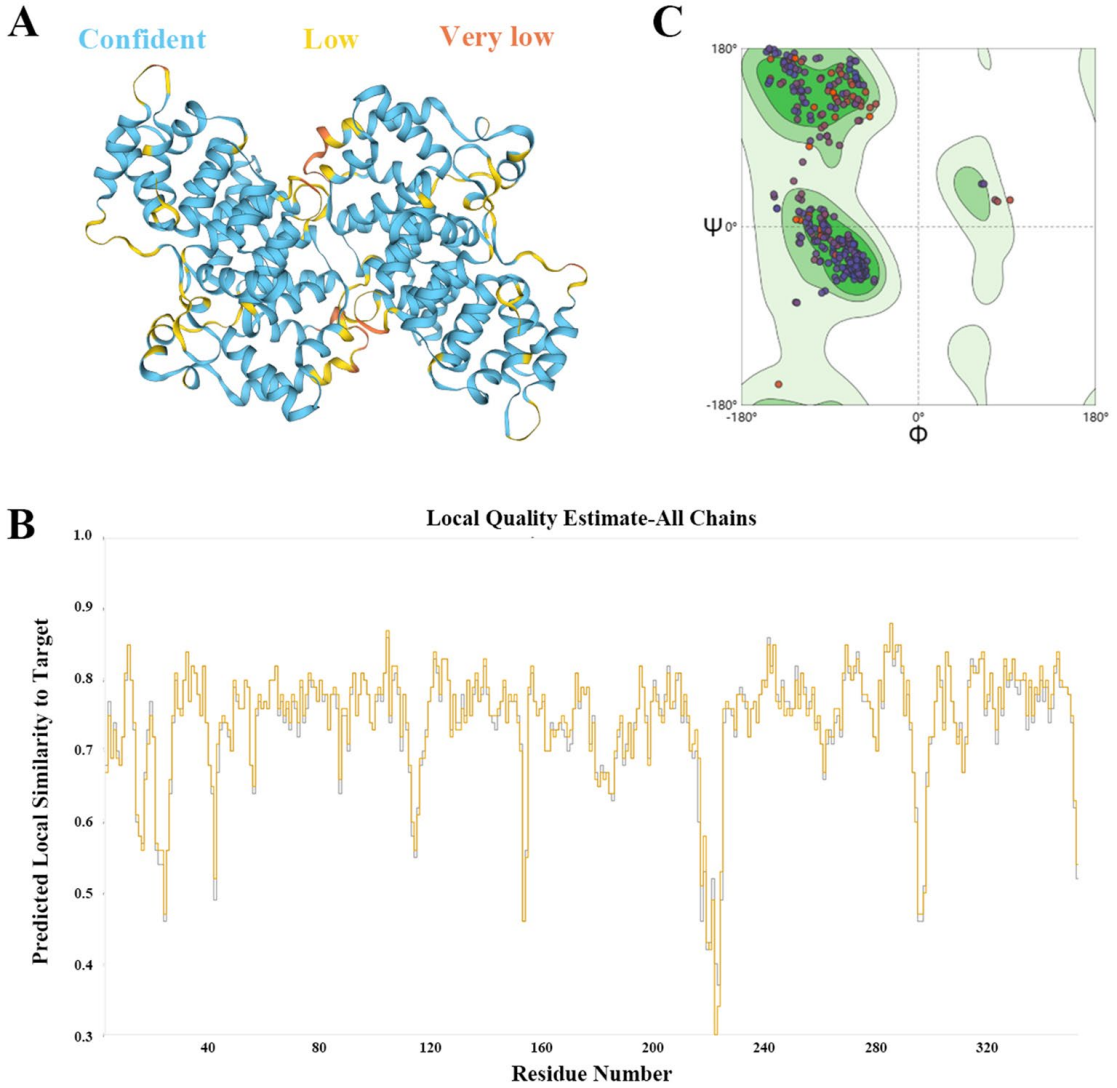
**Validation results of the refined 3D structure of SjANX A13. A** A 3D structure predicted for SjANX A13 (the structure was presented by “confidence” color, blue: confident, yellow: low, orange: very low) **B** The average per-residue QMEANDisCo score. **C** Ramachandran plot of the model showed that 96.85%, 0.29% and 0.49% of residues were located in the favored, allowed and disallowed regions, respectively.

The 3D structure of SjANX A13 was obtained based on the crystal structure of calcium-bound annexin (Sm)1 as a template (UniProt accession number: C4QH88). There was no significant difference in the conformation of the two molecules of the dimer (Figure 3A). The predicted model obtained with the SWISS-MODEL homology modelling pipeline had a Qualitative Model Energy Analysis-Distance Constraint (QMEANDisCo) global score of 0.75 ± 0.05 and Global Model Quality Estimation (GMQE) score of 0.78, where higher QMEANDisCo global and GMQE scores (range 0–1) indicate higher expected quality (Figure 3B). Ramachandran plot analysis revealed that the protein model had 96.85% of residues in most favored regions, 0.29% in allowed regions, and only 0.49% in disallowed regions (Figure 3C). Collectively, these findings indicate that the SjANX A13 model had correct topology with high expected quality.

### Bioinformatics analysis of SjANX A13

**Figure 4:**
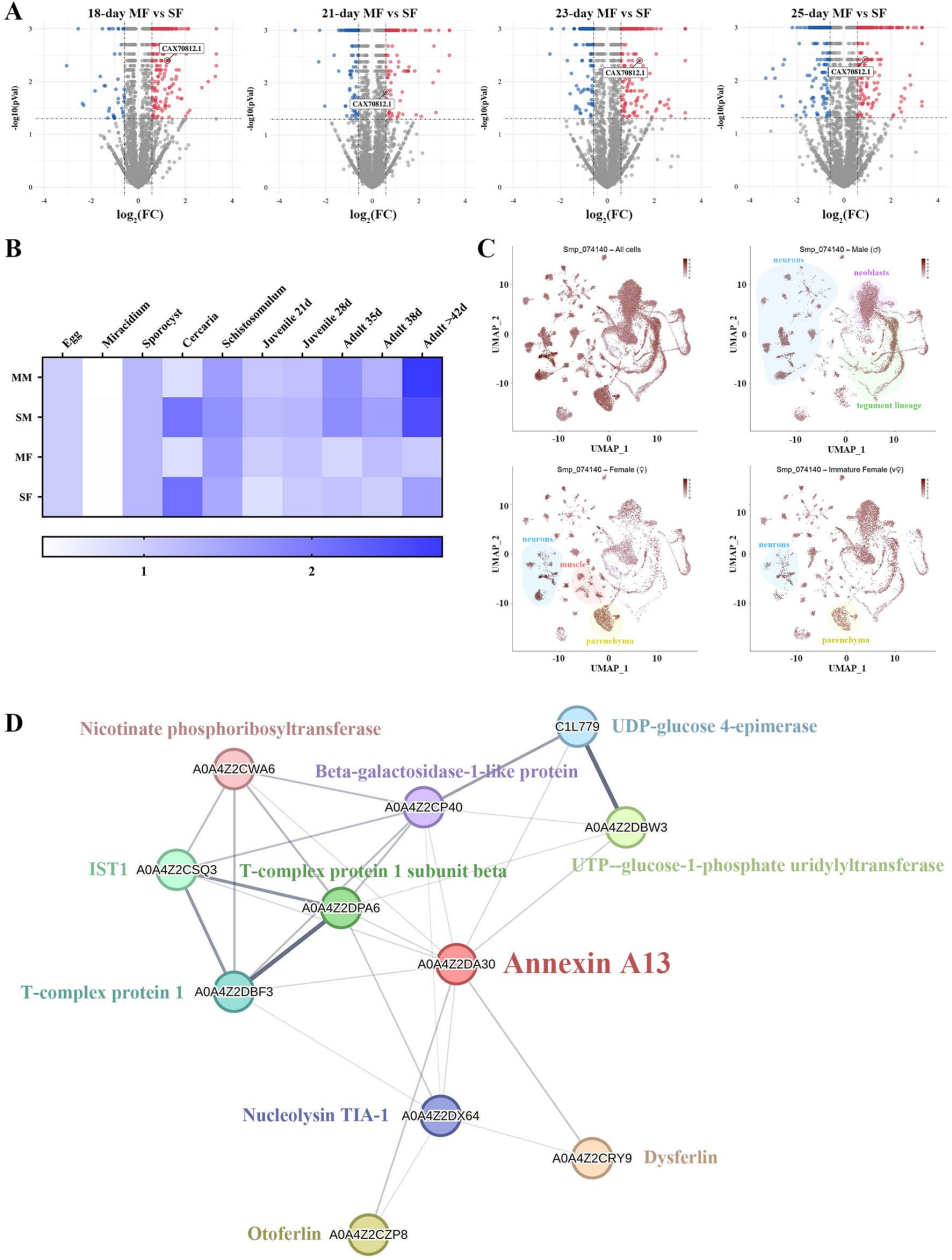
**Bioinformatics analysis of SjANX A13. A** Volcano plots showing the SjANX A13 protein expression in 18, 21, 23 and 25-day MF verses SF proteome. **B** A heatmap showing the SmANX (Smp_074140) gene expression during the schistosome life cycle. **C** ScRNAseq projections depicting the expression profiles of SmANX (Smp_074140) in different cell clusters in all cells, male, female and immature female worms of *S. mansoni* worms. **D** A network of SjANX A13 and its potential interacting proteins, and the line thickness indicates the strength of data support.

To obtain a comprehensive profile of SjANX A13 expression in the publicly available dataset, the proteomics data included in a previous report by our group was analyzed by mainly focusing on SF and MF worms at 18, 21, 23, and 25 days [17]. In this dataset, SjANX A13 expression was relatively higher in MF worms at all four time points, suggesting a potential role in female development (Figure 4A). In addition, the expression profiles of the *S. mansoni* homolog (Smp_074140) of SjANX A13 were analyzed in references to more well-established RNAseq [44] and scRNAseq [45] datasets. In terms of the life history of *S. mansoni*, SmANX (Smp_074140) was highly expressed in single-sex cercariae and mated male (MM) worms at 35 and > 42 days post-infection (dpi). In addition, Smp_074140 expression was generally higher in males than females at the same developmental time point, both in unisexual and bisexual infections (Figure 4B). Further comparison of unisexual- and bisexual-infected worms found that Smp_074140 expression was higher in SM than MM worms, with the exception of 42 dpi at the late maturation stage, while expression was generally higher in MF than SF worms, with the exception of 35 and 42 dpi, which was similar to the proteomic data of *S. japonicum* females mentioned above, as expression at 18–25 dpi in *S. japonicum* was almost identical to that at 28–35 dpi in *S. mansoni* [58] (Figure 4B).

Analysis of the scRNAseq data allowed assessment of the expression profiles of Smp_074140 in different cell types of *S. mansoni* worms. As shown in Figure 4C, Smp_074140 was enriched in almost all cell types. Smp_074140 was mainly enriched in the tegument lineage, neurons, and neoblasts cell clusters of MM worms, the neurons, muscle, and parenchyma cell clusters of MF worms, and relatively limited in the neurons and parenchyma of SF worms (Figure 4C).

A protein–protein interaction network that was constructed based on the STRING database (Figure 4D) identified 10 proteins that potentially interact with SjANX A13, which included beta-galactosidase-1-like protein, dysferlin, IST1, nicotinate phosphoribosyltransferase, otoferlin, T-complex protein 1, UTP-glucose-1-phosphate uridylyltransferase, T-complex protein 1 subunit beta, Nucleolysin TIA-1, and UDP-glucose 4-epimerase. SjANX A13 and predicted interacting proteins were enriched in the GO terms “chaperonin-containing T-complex” and “cytoplasm”, in addition to the KEGG pathway “galactose metabolism”, suggesting potential roles in cellular component and physiological processes (Additional file 2).

### Expression patterns of SjANX A13 at different time points after infection

**Figure 5:**
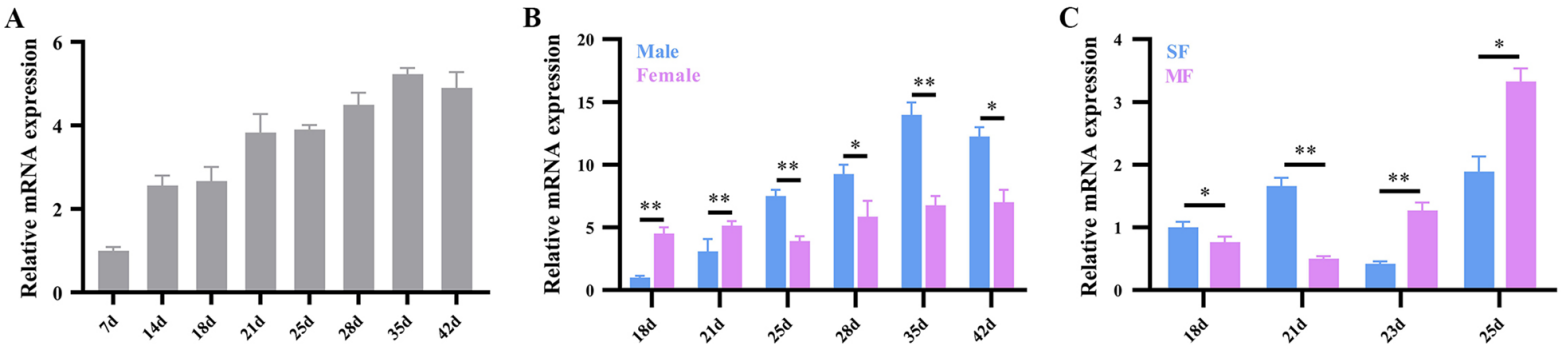
**Transcriptional profile of SjANX A13. A** Transcript levels of SjANX A13 in 7, 14, 18, 21, 25, 28, 35 and 42-day worms come from mixed cercariae-infected mice. **B** Transcript levels of SjANX A13 in 18, 21, 25, 28, 35 and 42-day mated male and female worms. **C** Transcript levels of SjANX A13 in 18, 21, 23 and 25-day SF and MF. All experiments were performed in triplicate and are expressed as the mean ± SD. Significant differences are indicated (*P < 0.05, **P < 0.01)

To further clarify the mRNA expression profiles of SjANX A13, schistosomes were recovered from infected mice at different time points after infection. The qPCR results revealed that the mRNA levels of SjANX A13 tended to increase at 7–42 dpi (Figure 5A). After mating (around 15 dpi for *S. japonicum*), SjANX A13 expression gradually increased in MM worms and surpassed that of females at 25 dpi, whereas SjANX A13 expression slightly fluctuated in MF worms at 18–42 dpi (Figure 5B). Such differences in expression pattern may indicate that SjANX A13 plays different roles in males and females during reproductive development. Further comparisons of SF and MF worms showed that SjANX A13 was highly expressed in SF worms before eggshell formation (around 21 dpi for *S. japonicum*), whereas after eggshell formation, SjANX A13 expression was significantly higher in MF than SF worms (Figure 5C).

### SjANX A13-knockdown affects the development and reproduction of *S. japonicum*

**Figure 6:**
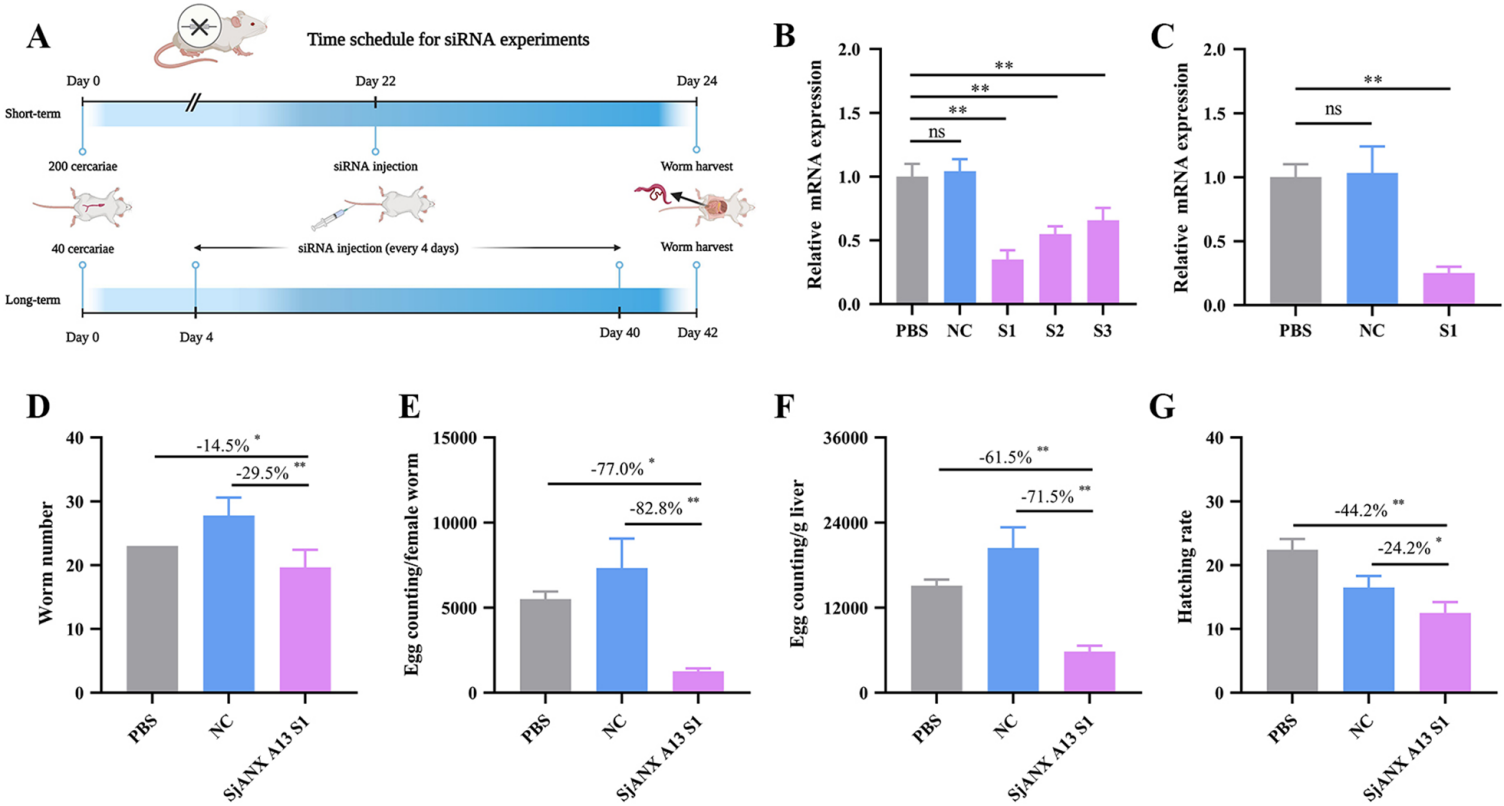
**SjANX A13 knockdown affects the vitality and reproduction of *S. japonicum*.** **A** Time schedule for siRNA efficiency assay and long-term siRNA experiment. **B** Comparison of the efficacies of the negative control and three SjANX A13-specific siRNA pairs. **C** Effects of SjANX A13 S1 siRNA on SjANX A13 gene expression after 10 injections between 4 and 42 dpi. **D** Effect of long-term siRNA treatment on the worm burden. **E** Effect of long-term siRNA treatment on reproductive capacity of female worms. **F** Effect of long-term siRNA treatment on the liver egg burden. **G** Effect of long-term siRNA treatment on egg hatching rate. All experiments were performed in triplicate and are expressed as the mean ± SD. Significant differences are indicated (*P < 0.05, **P < 0.01). The reduction rates are presented in the panels. **A** was created with Biorender.com.

RNAi was performed to determine the function of SjANX A13 in vivo. Three specific SjANX A13 siRNA pairs were first tested in vivo to identify the most efficient. At 22 dpi, siRNAs were injected via the tail vein and the parasites were harvested 48 h later (Figure 6A). The effects of gene silencing were determined by qPCR and S1 siRNA was chosen for further experiments (Figure 6B).

A long-term RNAi study revealed that SjANX A13 mRNA expression levels had significantly decreased after 10 injections with S1 siRNA, as detected by qPCR (Figures 6A and C). Morphological observations by SEM demonstrated that the MM and MF worms in the NC group had a continuous and well-organized network structure with clearly recognizable flower-like papillae. As compared to the NC group, most of the tegument of worms in the SjANX A13 S1 group showed no significant morphological changes, while the tegument surface of some areas appeared smoother (Additional file 3).

To further characterize the role of SjANX A13 in reproduction, the worm and liver egg burdens in mice were quantified. As compared to the PBS and NC groups, the number of worms and liver egg burden were reduced in the SjANX A13 S1 group by 14.5% and 29.5% (Figure 6D), while egg counts were decreased by 77.1% and 82.8%, respectively (Figure 6E). The loss of worms and reproductive capacity reduced the egg burden in the liver. As compared to the PBS and NC groups, the liver egg burden was decreased in the SjANX A13-knockdown group by 61.5% and 71.5%, respectively, while 44.2% and 24.2% of eggs harvested from the liver failed to hatch into miracidia (Figures 6F and G).

### Vaccine efficacy of rSjANX A13 against *S. japonicum*

**Figure 7:**
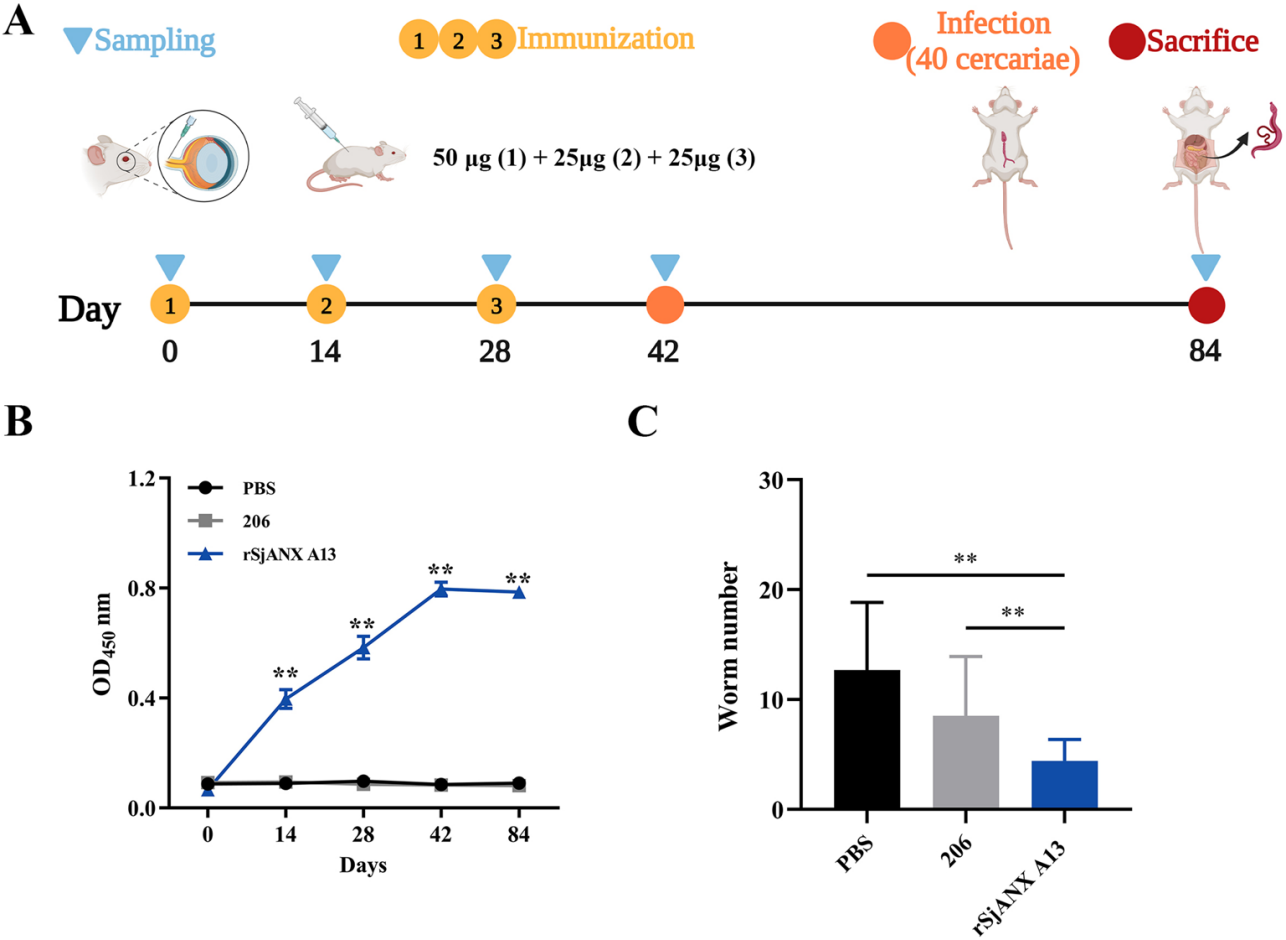
**Vaccine efficacy of rSjANX A13 against *S. japonicum*.** **A** Time schedule for immune protection assays. **B** Changes in IgG antibody levels. **C** Effect of rSjANX A13 immunization on the worm burden. All experiments were performed in triplicate and are expressed as the mean ± SD. Significant differences are indicated (ns > 0.05, **P < 0.01). **A** was created with Biorender.com.

To determine whether rSjANX A13 exhibits immunoprotective effects, the SjANX A13 gene was cloned into the plasmid pET28a(+) and successfully expressed in *E. coli* BL21 (DE3) cells. Following initiation of gene transcription with IPTG, the bacterial culture was briefly sonicated and the lysate was separated into soluble and insoluble fractions. Most of the recombinant protein was contained in the supernatant and then purified by affinity chromatography using a His-tag purification column. The purified rSjANX A13 protein was verified by SDS–PAGE and stained with Coomassie brilliant blue, which showed a single band of the expected size (Additional file 4). Then, the immunoprotective effects of rSjANX A13 were assessed with an immune protection assay (Figure 7A). Serum levels of IgG-specific antibodies against rSjANX A13 of immunized and control mice were measured with an ELISA. The results showed that rSjANX A13 induced increased production of IgG-specific antibodies, with extremely elevated levels after the first immunization (Figure 7B). During the second and third subsequent immunizations, antibody levels were increased as compared to the first immunization and remained at high levels for 2 weeks after immunization (Figure 7B). Unfortunately, however, perhaps due to the imbalanced sex ratio of cercariae, which resulted in the collection of fewer female worms and complicated calculation of the liver egg burden, only the rate of worm reduction could be calculated, and the results showed that the worm burden was reduced (Figure 7C). However, this assay should be repeated because the infection process may have been flawed.

### SjANX A13 participates in host–parasite interactions via exosomes

**Figure 8:**
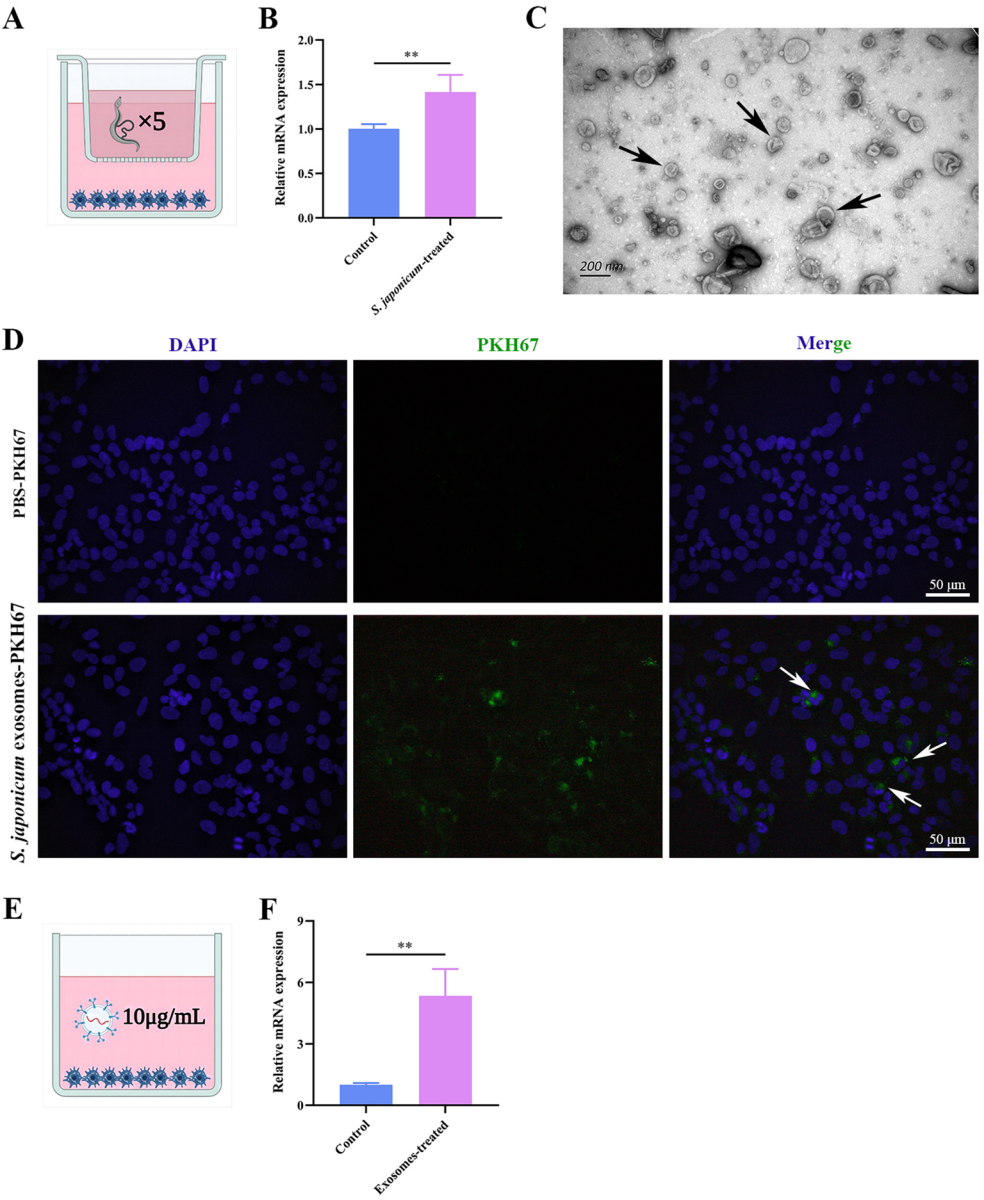
**SjANX A13 is involved in the host–parasite interaction via exosomes. A** Schema of the transwell system. **B** Transcript levels of SjANX A13 in LX-2 cells of the transwell system. **C** Observation of *S. japonicum* worm-derived exosomes through TEM. The white arrows highlight typical structure of exosomes. Scale bar = 200 nm. **D** Representative images of the uptake of PKH67-labeled *S. japonicum* worm-derived exosomes (green) by LX-2 cells (DAPI blue). The black arrows highlight representative internalization. Scale bar = 50 μm. **E** Schema of exosomes and LX-2 cells co-incubation. **F** Transcript levels of SjANX A13 in LX-2 cells after coculture with *S. japonicum* worm-derived exosomes after 48 h. **A** and **E** were created with Biorender.com.

Previous studies have shown that SjANX A13 is an important component of the excretory/secretory cargo of the exosomes of *S*. *japonicum* [32, 33]. To determine whether SjANX A13 is involved in host–parasite interactions through exosomes, a transwell model was constructed to simulate the activities of *S. japonicum* in host cells (Figure 8A). Following co-culture for 48 h, the presence of the SjANX A13 gene in LX-2 cells was verified by qPCR (Figure 8B), suggesting that *S. japonicum* worms can be delivered into host cells via exosomes. Thus, *S. japonicum*-derived exosomes were isolated from host cells as described in previous studies [33, 57, 59]. As shown in Figure 8C, TEM analysis confirmed enrichment of exosomes with the typical cup-like morphology. Next, the ability of worm-derived exosomes to deliver A13 directly into host cells was investigated. The worm-derived exosomes were purified and labeled with a green fluorescent dye (PKH67). Uptake of the PKH67-labeled exosomes by LX-2 cells was verified by fluorescence microscopy. The green fluorescence of worm-derived exosomes labeled with PKH67 was observed in LX-2 cells, demonstrating uptake of worm-derived exosomes by LX-2 cells, while LX-2 cells co-cultured with PKH67-stained PBS showed no green fluorescence (Figure 8D). Subsequently, exosome proteins at 10 µg/mL were selected for subsequent in vitro studies (Figure 8E). The results of qPCR analysis verified the presence of SjANX A13 in LX-2 cells (Figure 8F). Collectively, these results suggest that SjANX A13 might be involved in host–parasite interactions via exosomes.

## Discussion

Despite the similarities in amino acid sequences among vertebrates, invertebrates, fungi, plants, and protists, ANXs of eukaryotes have unique structural features and biological functions. The current nomenclature classifies ANXs as “A” for vertebrates, “B” for invertebrates (including schistosomes), “C” for fungi, “D” for plants, and “E” for protists [60]. With the recent and rapid updates of high-throughput databases for genomic and transcriptomics studies of parasitic helminths, there have been some inconsistencies in the nomenclature of ANXs in group B [61]. For instance, SjANX A13 is currently classified as group A (vertebrates) rather than group B. Therefore, the current nomenclature of SjANX A13 was maintained in the present study. However, a rational and consistent nomenclature is required to reflect the biological roles of ANXs.

Structurally, ANXs are characterized by similar C-terminal structural domains that consist of four homologous repeats of approximately 70 amino acids each, which have similar functions [61]. Recent studies have revealed that several ANXs, such as SmANX B22 of *S. mansoni* [29] and SbANX of *S. bovis* [26], are tegument components. In the present study, the structural domain composition of SjANX A13 was analyzed in reference to the SMART website, which showed that each homologous repeat was composed of approximately 60 amino acids. Meanwhile, the potential expression patterns of SjANX A13 were explored in reference to SmANX (Smp_074140), which has the highest homology (74.01%) with SjANX A13, and the scRNAseq database. According to the *S. mansoni* scRNAseq profile, Smp_074140 was only slightly expressed in the tegument cells of MM worms, which were not the predominant cell type. Although only the *S. mansoni* database was used as a reference, in conjunction with the results of the SjANX A13 RNAi assay in the present study, the lack of significant changes to the tegument after SjANX A13-knockdown in MM worms also suggests that SjANX A13 is not mainly expressed in tegument cells. In fact, mouse ANX A13 is an intestine-specific protein that is localized at the tips of the intestinal microvilli [62].

To further explore the function of SjANX A13, it was determined that the highest expression of Smp_074140 occurs in neurons, which are essential to the reproductive development of schistosomes [63]. In addition, the high expression of SjANX A13 in MF worms at 18, 21, 23, and 25 days also suggests potential roles in the growth and development of *S. japonicum* [17]. In the present study, long-term RNAi of SjANX A13 significantly reduced the fecundity of female worms and hatch capacity of eggs, indicating the involvement of SjANX A13 in the development of *S. japonicum.* Moreover, a protein-protein interaction network was constructed to identify proteins that potentially interact with SjANX A13. Among the identified proteins, expression of UDP-glucose 4-epimerase [64] and dysferlin [65] was mainly limited to the tegument cells of *S. japonicum* and a recombinant protein of UDP-glucose 4-epimerase and dysferlin showed potential as a vaccine target. Besides, T-complex protein 1 is involved cytoskeletal proteostasis [66] and nucleolysin TIA-1 is critical to the stress response and neurodevelopment in mammals [67]. Nonetheless, further studies are needed to determine whether SjANX A13 is related to the functions of these proteins.

Several ANXs located at the schistosome tegument interface with immunogenic properties have been proposed as potential vaccine candidates [24, 68]. In the present study, the immunoprotective effects of rSjANX A13 were evaluated. Due to the possible imbalance between male and female cercariae used in the assay, it was not possible to determine the extent of the liver egg burden, even though the worm burden was reduced. Therefore, the experiments must be repeated to further verify the results.

In addition to the potential biological activities against schistosomes, the effects of SjANX A13 as an excretory/secretory protein on the host cell were explored [32]. Notably, SbANX of *S. bovis* exhibits fibrinolytic and anticoagulant activities [26]. Recently, SjANX A13 was also identified in *S. japonicum* exosomes, which are considered important vectors for the regulation of host–parasite interactions [33, 69]. In the present study, through the construction of a transwell system and co-culture *S. japonicum* exosomes with LX-2, SjANX A13 was detected in host cells, suggesting participation in host–parasite interactions through exosomes.

In conclusion, based on the results of a previous proteomic study conducted by our group, bioinformatics, RNAi, and qPCR analyses were used to assess the potential involvement of SjANX A13 in the reproductive development of *S. japonicum*. The results confirmed the involvement of SjANX A13 in the host–parasite interactions via exosomes. However, further investigations are needed to assess the suitability of SjANX A13 as a vaccine candidate.

### Supplementary Information

**Additional file 1. Signal peptide and transmembrane region analysis of SjANX A13.** **A** Analysis of signal peptide structure of SjANX A13. **B** Transmembrane region analysis of SjANX A13.

**Additional file 2. Functional analysis of SjANX A13 and its potential interacting molecules.**

**Additional file 3. Morphological observation through SEM after long-term SjANX A13 knockdown. **MM and MF worms from NC group had a continuous and well-organized network structure with clearly recognizable flower-like papillae, compared with the NC group, most of the tegument from worms of SjANX A13 S1 group showed no significant morphological changes, while the tegument in some areas (head part of male and middle part of female) appeared a smoother surface. ns: network structure, flp: fower-like papillae, b: bubbles, p: protrusion, pmp: pedicle mastoid process. Scale bar = 10 μm.

**Additional file 4. Amplification of SjANX A13 gene digested with vector pET-28a(+) and SDS-PAGE analysis of the expression of recombinant SjANX A13 protein.** **A** Amplification of SjANX A13. Lane M: DL 2000 DNA marker; Lanes 1 and 2, PCR product of target gene SjANX A13. **B** Lane M: protein marker; Lanes 1, 2, 3, 4 and 5 are 0 h, 2 h, 4 h, 6 h, 8 h of rSjANX A13 induction, respectively. **C** SDS-PAGE detection of recombinant SjANX A13 protein (Coomassie). Lane M: protein marker; Lane 1: r SjANX A13 induced supernatant; Lane 2: r SjANX A13 induced precipitation; Lane 3: r SjANX A13 recombinant protein purified product.

## Data Availability

The datasets used or analyzed during the current study are available from the corresponding author on reasonable request.
