# Supplementary figures and images for: Molecular and functional characterization of Schistosoma japonicum annexin A13
Source: Vet Res. 2023 Dec 4;54:116. doi: 10.1186/s13567-023-01244-z (PMC10696758; doi:10.1186/s13567-023-01244-z)

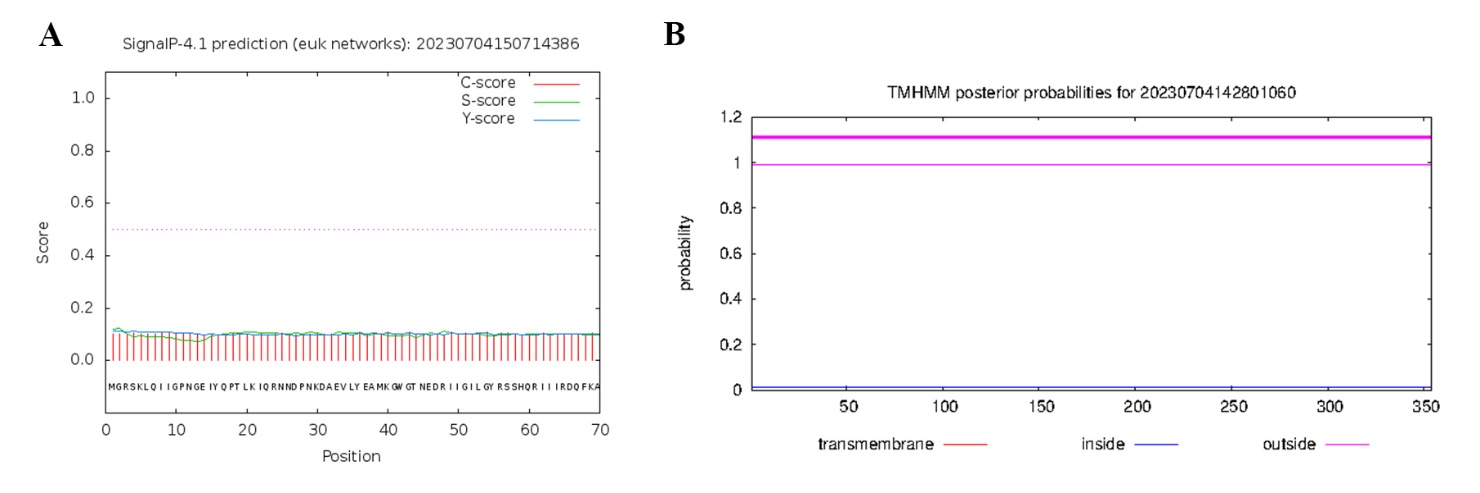

Supplement: Supplementary file 1 — Additional file 1. Signal peptide and transmembrane region analysis of SjANX A13. A Analysis of signal peptide structure of SjANX A13. B Transmembrane region analysis of SjANX A13. [file 13567_2023_1244_MOESM1_ESM.docx]

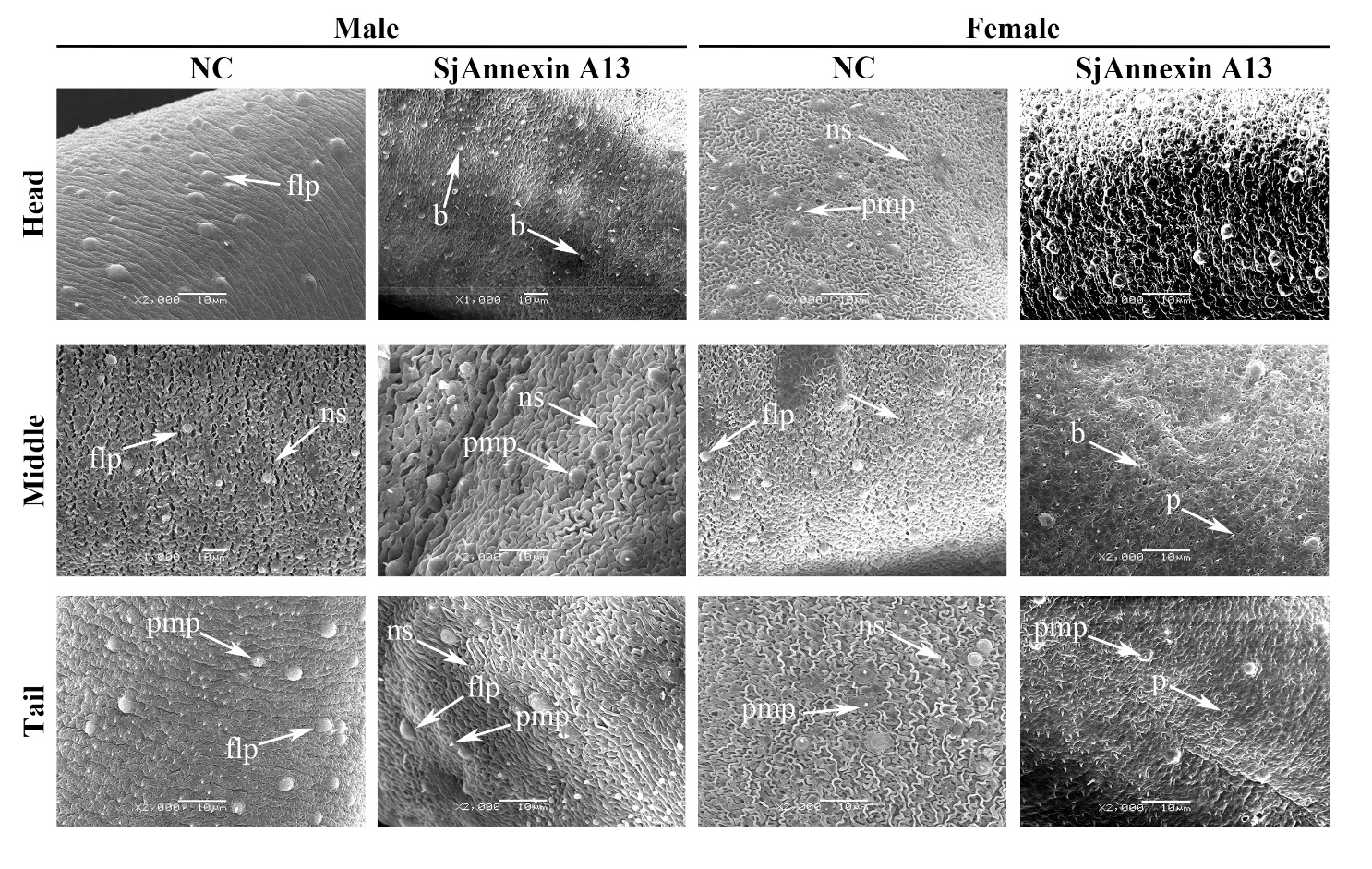

Supplement: Supplementary file 3 — Additional file 3. Morphological observation through SEM after long-term SjANX A13 knockdown. MM and MF worms from NC group had a continuous and well-organized network structure with clearly recognizable flower-like papillae, compared with the NC group, most of the tegument from worms of SjANX A13 S1 group showed no significant morphological changes, while the tegument in some areas (head part of male and middle part of female) appeared a smoother surface. ns: network structure, flp: fower-like papillae, b: bubbles, p: protrusion, pmp: pedicle mastoid process. Scale bar = 10 μm. [file 13567_2023_1244_MOESM3_ESM.docx]

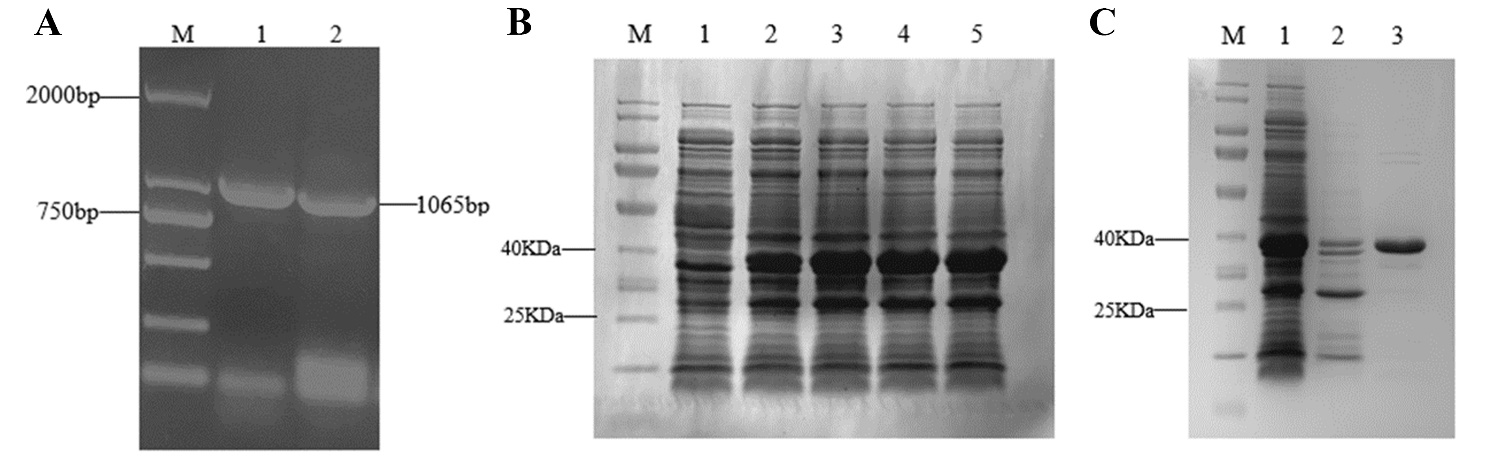

Supplement: Supplementary file 4 — Additional file 4. Amplification of SjANX A13 gene digested with vector pET-28a(+) and SDS-PAGE analysis of the expression of recombinant SjANX A13 protein. A Amplification of SjANX A13. Lane M: DL 2000 DNA marker; Lanes 1 and 2, PCR product of target gene SjANX A13. B Lane M: protein marker; Lanes 1, 2, 3, 4 and 5 are 0 h, 2 h, 4 h, 6 h, 8 h of rSjANX A13 induction, respectively. C SDS-PAGE detection of recombinant SjANX A13 protein (Coomassie). Lane M: protein marker; Lane 1: r SjANX A13 induced supernatant; Lane 2: r SjANX A13 induced precipitation; Lane 3: r SjANX A13 recombinant protein purified product. [file 13567_2023_1244_MOESM4_ESM.docx]
