# Supplementary material for: Molecular and functional characterization of Schistosoma japonicum annexin A13
Source: Vet Res. 2023 Dec 4;54:116. doi: 10.1186/s13567-023-01244-z (PMC10696758; doi:10.1186/s13567-023-01244-z)
Supplement: Supplementary file 2 — Additional file 2. Functional analysis of SjANX A13 and its potential interacting molecules. [file 13567_2023_1244_MOESM2_ESM.docx]

**Additional file 2** Functional analysis of SjANX A13 and its potential interacting molecules

| Name | #Node | **Category** | **Term description** |
| --- | --- | --- | --- |
| Beta-galactosidase-1-like protein isoform 1 | A0A4Z2CP40 | GO Process | Carbohydrate metabolic process |
|  | A0A4Z2CP40 | GO Process | Metabolic process |
|  | A0A4Z2CP40 | GO Process | Primary metabolic process |
|  | A0A4Z2CP40 | GO Process | Organic substance metabolic process |
|  | A0A4Z2CP40 | GO Function | Catalytic activity |
|  | A0A4Z2CP40 | GO Function | Hydrolase activity, hydrolyzing O-glycosyl compounds |
|  | A0A4Z2CP40 | GO Function | Beta-galactosidase activity |
|  | A0A4Z2CP40 | GO Function | Galactosidase activity |
|  | A0A4Z2CP40 | GO Function | Hydrolase activity |
|  | A0A4Z2CP40 | GO Function | Hydrolase activity, acting on glycosyl bonds |
|  | A0A4Z2CP40 | GO Component | Intracellular |
|  | A0A4Z2CP40 | GO Component | Cytoplasm |
|  | A0A4Z2CP40 | GO Component | Vacuole |
|  | A0A4Z2CP40 | GO Component | Organelle |
|  | A0A4Z2CP40 | GO Component | Membrane-bounded organelle |
|  | A0A4Z2CP40 | GO Component | Intracellular organelle |
|  | A0A4Z2CP40 | GO Component | Intracellular membrane-bounded organelle |
|  | A0A4Z2CP40 | GO Component | Cellular anatomical entity |
|  | A0A4Z2CP40 | KEGG | Galactose metabolism |
|  | A0A4Z2CP40 | KEGG | Other glycan degradation |
|  | A0A4Z2CP40 | KEGG | Glycosaminoglycan degradation |
|  | A0A4Z2CP40 | KEGG | Sphingolipid metabolism |
|  | A0A4Z2CP40 | KEGG | Glycosphingolipid biosynthesis - ganglio series |
|  | A0A4Z2CP40 | KEGG | Metabolic pathways |
|  | A0A4Z2CP40 | KEGG | Lysosome |
| Dysferlin | A0A4Z2CRY9 | GO Process | Plasma membrane organization |
|  | A0A4Z2CRY9 | GO Process | Cellular process |
|  | A0A4Z2CRY9 | GO Process | Endomembrane system organization |
|  | A0A4Z2CRY9 | GO Process | Cellular component organization |
|  | A0A4Z2CRY9 | GO Process | Membrane organization |
|  | A0A4Z2CRY9 | GO Process | Biological regulation |
|  | A0A4Z2CRY9 | GO Process | Cellular component organization or biogenesis |
|  | A0A4Z2CRY9 | GO Function | Binding |
|  | A0A4Z2CRY9 | GO Function | Ion binding |
|  | A0A4Z2CRY9 | GO Function | Cation binding |
|  | A0A4Z2CRY9 | GO Function | Metal ion binding |
|  | A0A4Z2CRY9 | GO Component | Intracellular |
|  | A0A4Z2CRY9 | GO Component | Cytoplasm |
|  | A0A4Z2CRY9 | GO Component | Plasma membrane |
|  | A0A4Z2CRY9 | GO Component | Membrane |
|  | A0A4Z2CRY9 | GO Component | Integral component of membrane |
|  | A0A4Z2CRY9 | GO Component | Organelle membrane |
|  | A0A4Z2CRY9 | GO Component | Intrinsic component of membrane |
|  | A0A4Z2CRY9 | GO Component | Cytoplasmic vesicle |
|  | A0A4Z2CRY9 | GO Component | Vesicle |
|  | A0A4Z2CRY9 | GO Component | Organelle |
|  | A0A4Z2CRY9 | GO Component | Membrane-bounded organelle |
|  | A0A4Z2CRY9 | GO Component | Intracellular organelle |
|  | A0A4Z2CRY9 | GO Component | Cell periphery |
|  | A0A4Z2CRY9 | GO Component | Intracellular vesicle |
|  | A0A4Z2CRY9 | GO Component | Cellular anatomical entity |
| IST1 | A0A4Z2CSQ3 | GO Process | Transport |
|  | A0A4Z2CSQ3 | GO Process | Protein localization |
|  | A0A4Z2CSQ3 | GO Process | Cellular process |
|  | A0A4Z2CSQ3 | GO Process | Protein transport |
|  | A0A4Z2CSQ3 | GO Process | Peptide transport |
|  | A0A4Z2CSQ3 | GO Process | Macromolecule localization |
|  | A0A4Z2CSQ3 | GO Process | Amide transport |
|  | A0A4Z2CSQ3 | GO Process | Establishment of protein localization |
|  | A0A4Z2CSQ3 | GO Process | Localization |
|  | A0A4Z2CSQ3 | GO Process | Establishment of localization |
|  | A0A4Z2CSQ3 | GO Process | Organic substance transport |
|  | A0A4Z2CSQ3 | GO Process | Nitrogen compound transport |
|  | A0A4Z2CSQ3 | GO Function | Binding |
|  | A0A4Z2CSQ3 | GO Function | Protein binding |
|  | A0A4Z2CSQ3 | GO Function | Protein domain specific binding |
|  | A0A4Z2CSQ3 | GO Function | Identical protein binding |
|  | A0A4Z2CSQ3 | GO Function | Protein-containing complex binding |
|  | A0A4Z2CSQ3 | GO Component | Intracellular |
|  | A0A4Z2CSQ3 | GO Component | Nucleus |
|  | A0A4Z2CSQ3 | GO Component | Nuclear envelope |
|  | A0A4Z2CSQ3 | GO Component | Cytoplasm |
|  | A0A4Z2CSQ3 | GO Component | Microtubule organizing center |
|  | A0A4Z2CSQ3 | GO Component | Cytoskeleton |
|  | A0A4Z2CSQ3 | GO Component | Endomembrane system |
|  | A0A4Z2CSQ3 | GO Component | Microtubule cytoskeleton |
|  | A0A4Z2CSQ3 | GO Component | Midbody |
|  | A0A4Z2CSQ3 | GO Component | Cytoplasmic vesicle |
|  | A0A4Z2CSQ3 | GO Component | Organelle envelope |
|  | A0A4Z2CSQ3 | GO Component | Envelope |
|  | A0A4Z2CSQ3 | GO Component | Vesicle |
|  | A0A4Z2CSQ3 | GO Component | Organelle |
|  | A0A4Z2CSQ3 | GO Component | Membrane-bounded organelle |
|  | A0A4Z2CSQ3 | GO Component | Non-membrane-bounded organelle |
|  | A0A4Z2CSQ3 | GO Component | Intracellular organelle |
|  | A0A4Z2CSQ3 | GO Component | Intracellular membrane-bounded organelle |
|  | A0A4Z2CSQ3 | GO Component | Intracellular non-membrane-bounded organelle |
|  | A0A4Z2CSQ3 | GO Component | Intracellular vesicle |
|  | A0A4Z2CSQ3 | GO Component | Cellular anatomical entity |
|  | A0A4Z2CSQ3 | KEGG | Endocytosis |
| Nicotinate phosphoribosyltransferase | A0A4Z2CWA6 | GO Process | Nucleobase-containing compound metabolic process |
|  | A0A4Z2CWA6 | GO Process | Cellular aromatic compound metabolic process |
|  | A0A4Z2CWA6 | GO Process | Nucleoside phosphate metabolic process |
|  | A0A4Z2CWA6 | GO Process | Phosphorus metabolic process |
|  | A0A4Z2CWA6 | GO Process | Phosphate-containing compound metabolic process |
|  | A0A4Z2CWA6 | GO Process | Nitrogen compound metabolic process |
|  | A0A4Z2CWA6 | GO Process | Response to stress |
|  | A0A4Z2CWA6 | GO Process | Response to oxidative stress |
|  | A0A4Z2CWA6 | GO Process | Metabolic process |
|  | A0A4Z2CWA6 | GO Process | Biosynthetic process |
|  | A0A4Z2CWA6 | GO Process | Nucleotide metabolic process |
|  | A0A4Z2CWA6 | GO Process | Nucleotide biosynthetic process |
|  | A0A4Z2CWA6 | GO Process | NAD biosynthetic process |
|  | A0A4Z2CWA6 | GO Process | Cellular process |
|  | A0A4Z2CWA6 | GO Process | Heterocycle biosynthetic process |
|  | A0A4Z2CWA6 | GO Process | Nicotinamide nucleotide biosynthetic process |
|  | A0A4Z2CWA6 | GO Process | Pyridine nucleotide metabolic process |
|  | A0A4Z2CWA6 | GO Process | Pyridine nucleotide biosynthetic process |
|  | A0A4Z2CWA6 | GO Process | Pyridine nucleotide salvage |
|  | A0A4Z2CWA6 | GO Process | Aromatic compound biosynthetic process |
|  | A0A4Z2CWA6 | GO Process | Organophosphate metabolic process |
|  | A0A4Z2CWA6 | GO Process | NAD salvage |
|  | A0A4Z2CWA6 | GO Process | Cellular nitrogen compound metabolic process |
|  | A0A4Z2CWA6 | GO Process | Nucleobase-containing compound biosynthetic process |
|  | A0A4Z2CWA6 | GO Process | Cellular metabolic compound salvage |
|  | A0A4Z2CWA6 | GO Process | Nucleotide salvage |
|  | A0A4Z2CWA6 | GO Process | Cellular metabolic process |
|  | A0A4Z2CWA6 | GO Process | Primary metabolic process |
|  | A0A4Z2CWA6 | GO Process | Cellular biosynthetic process |
|  | A0A4Z2CWA6 | GO Process | Cellular nitrogen compound biosynthetic process |
|  | A0A4Z2CWA6 | GO Process | Small molecule metabolic process |
|  | A0A4Z2CWA6 | GO Process | Heterocycle metabolic process |
|  | A0A4Z2CWA6 | GO Process | Nicotinamide nucleotide metabolic process |
|  | A0A4Z2CWA6 | GO Process | Response to stimulus |
|  | A0A4Z2CWA6 | GO Process | Nucleobase-containing small molecule metabolic process |
|  | A0A4Z2CWA6 | GO Process | Organic substance metabolic process |
|  | A0A4Z2CWA6 | GO Process | Pyridine-containing compound metabolic process |
|  | A0A4Z2CWA6 | GO Process | Pyridine-containing compound biosynthetic process |
|  | A0A4Z2CWA6 | GO Process | Organophosphate biosynthetic process |
|  | A0A4Z2CWA6 | GO Process | Nucleoside phosphate biosynthetic process |
|  | A0A4Z2CWA6 | GO Process | Organic cyclic compound metabolic process |
|  | A0A4Z2CWA6 | GO Process | Organic cyclic compound biosynthetic process |
|  | A0A4Z2CWA6 | GO Process | Organonitrogen compound metabolic process |
|  | A0A4Z2CWA6 | GO Process | Organonitrogen compound biosynthetic process |
|  | A0A4Z2CWA6 | GO Process | Organic substance biosynthetic process |
|  | A0A4Z2CWA6 | GO Function | Catalytic activity |
|  | A0A4Z2CWA6 | GO Function | Nicotinate-nucleotide diphosphorylase (carboxylating) activity |
|  | A0A4Z2CWA6 | GO Function | Nicotinate phosphoribosyltransferase activity |
|  | A0A4Z2CWA6 | GO Function | Transferase activity |
|  | A0A4Z2CWA6 | GO Function | Transferase activity, transferring glycosyl groups |
|  | A0A4Z2CWA6 | GO Function | Transferase activity, transferring pentosyl groups |
|  | A0A4Z2CWA6 | GO Function | Ligase activity |
|  | A0A4Z2CWA6 | GO Function | Ligase activity, forming carbon-nitrogen bonds |
|  | A0A4Z2CWA6 | GO Component | Intracellular |
|  | A0A4Z2CWA6 | GO Component | Cytoplasm |
|  | A0A4Z2CWA6 | GO Component | Cytosol |
|  | A0A4Z2CWA6 | GO Component | Cellular anatomical entity |
|  | A0A4Z2CWA6 | KEGG | Nicotinate and nicotinamide metabolism |
|  | A0A4Z2CWA6 | KEGG | Metabolic pathways |
| Otoferlin | A0A4Z2CZP8 | GO Process | Plasma membrane organization |
|  | A0A4Z2CZP8 | GO Process | Cellular process |
|  | A0A4Z2CZP8 | GO Process | Endomembrane system organization |
|  | A0A4Z2CZP8 | GO Process | Cellular component organization |
|  | A0A4Z2CZP8 | GO Process | Membrane organization |
|  | A0A4Z2CZP8 | GO Process | Biological regulation |
|  | A0A4Z2CZP8 | GO Process | Cellular component organization or biogenesis |
|  | A0A4Z2CZP8 | GO Function | Binding |
|  | A0A4Z2CZP8 | GO Function | Ion binding |
|  | A0A4Z2CZP8 | GO Function | Cation binding |
|  | A0A4Z2CZP8 | GO Function | Metal ion binding |
|  | A0A4Z2CZP8 | GO Component | Intracellular |
|  | A0A4Z2CZP8 | GO Component | Cytoplasm |
|  | A0A4Z2CZP8 | GO Component | Plasma membrane |
|  | A0A4Z2CZP8 | GO Component | Membrane |
|  | A0A4Z2CZP8 | GO Component | Integral component of membrane |
|  | A0A4Z2CZP8 | GO Component | Organelle membrane |
|  | A0A4Z2CZP8 | GO Component | Intrinsic component of membrane |
|  | A0A4Z2CZP8 | GO Component | Cytoplasmic vesicle |
|  | A0A4Z2CZP8 | GO Component | Vesicle |
|  | A0A4Z2CZP8 | GO Component | Organelle |
|  | A0A4Z2CZP8 | GO Component | Membrane-bounded organelle |
|  | A0A4Z2CZP8 | GO Component | Intracellular organelle |
|  | A0A4Z2CZP8 | GO Component | Cell periphery |
|  | A0A4Z2CZP8 | GO Component | Intracellular vesicle |
|  | A0A4Z2CZP8 | GO Component | Cellular anatomical entity |
| Annexin A13 | A0A4Z2DA30 | GO Process | Cellular process |
|  | A0A4Z2DA30 | GO Function | Binding |
|  | A0A4Z2DA30 | GO Function | Calcium ion binding |
|  | A0A4Z2DA30 | GO Function | Protein binding |
|  | A0A4Z2DA30 | GO Function | Phospholipid binding |
|  | A0A4Z2DA30 | GO Function | Calcium-dependent phospholipid binding |
|  | A0A4Z2DA30 | GO Function | Lipid binding |
|  | A0A4Z2DA30 | GO Function | Ion binding |
|  | A0A4Z2DA30 | GO Function | Anion binding |
|  | A0A4Z2DA30 | GO Function | Cation binding |
|  | A0A4Z2DA30 | GO Function | Metal ion binding |
|  | A0A4Z2DA30 | GO Component | Intracellular |
|  | A0A4Z2DA30 | GO Component | Cytoplasm |
|  | A0A4Z2DA30 | GO Component | Endomembrane system |
|  | A0A4Z2DA30 | GO Component | Vesicle |
|  | A0A4Z2DA30 | GO Component | Organelle |
|  | A0A4Z2DA30 | GO Component | Membrane-bounded organelle |
|  | A0A4Z2DA30 | GO Component | Intracellular organelle |
|  | A0A4Z2DA30 | GO Component | Intracellular membrane-bounded organelle |
|  | A0A4Z2DA30 | GO Component | Cellular anatomical entity |
| T-complex protein 1 | A0A4Z2DBF3 | GO Process | Protein folding |
|  | A0A4Z2DBF3 | GO Process | Cellular process |
|  | A0A4Z2DBF3 | GO Function | Nucleotide binding |
|  | A0A4Z2DBF3 | GO Function | Binding |
|  | A0A4Z2DBF3 | GO Function | Protein binding |
|  | A0A4Z2DBF3 | GO Function | ATP binding |
|  | A0A4Z2DBF3 | GO Function | Purine nucleotide binding |
|  | A0A4Z2DBF3 | GO Function | Adenyl nucleotide binding |
|  | A0A4Z2DBF3 | GO Function | Ribonucleotide binding |
|  | A0A4Z2DBF3 | GO Function | Purine ribonucleotide binding |
|  | A0A4Z2DBF3 | GO Function | Adenyl ribonucleotide binding |
|  | A0A4Z2DBF3 | GO Function | Purine ribonucleoside triphosphate binding |
|  | A0A4Z2DBF3 | GO Function | Small molecule binding |
|  | A0A4Z2DBF3 | GO Function | Ion binding |
|  | A0A4Z2DBF3 | GO Function | Anion binding |
|  | A0A4Z2DBF3 | GO Function | Unfolded protein binding |
|  | A0A4Z2DBF3 | GO Function | Organic cyclic compound binding |
|  | A0A4Z2DBF3 | GO Function | Carbohydrate derivative binding |
|  | A0A4Z2DBF3 | GO Function | Nucleoside phosphate binding |
|  | A0A4Z2DBF3 | GO Function | Heterocyclic compound binding |
|  | A0A4Z2DBF3 | GO Component | Intracellular |
|  | A0A4Z2DBF3 | GO Component | Cytoplasm |
|  | A0A4Z2DBF3 | GO Component | Cytosol |
|  | A0A4Z2DBF3 | GO Component | Chaperonin-containing T-complex |
|  | A0A4Z2DBF3 | GO Component | Protein-containing complex |
|  | A0A4Z2DBF3 | GO Component | Chaperone complex |
|  | A0A4Z2DBF3 | GO Component | Cellular anatomical entity |
| UTP--glucose-1-phosphate uridylyltransferase | A0A4Z2DBW3 | GO Process | Carbohydrate metabolic process |
|  | A0A4Z2DBW3 | GO Process | Polysaccharide metabolic process |
|  | A0A4Z2DBW3 | GO Process | Glycogen metabolic process |
|  | A0A4Z2DBW3 | GO Process | UDP-glucose metabolic process |
|  | A0A4Z2DBW3 | GO Process | Cellular glucan metabolic process |
|  | A0A4Z2DBW3 | GO Process | Generation of precursor metabolites and energy |
|  | A0A4Z2DBW3 | GO Process | Energy reserve metabolic process |
|  | A0A4Z2DBW3 | GO Process | Nucleobase-containing compound metabolic process |
|  | A0A4Z2DBW3 | GO Process | Cellular aromatic compound metabolic process |
|  | A0A4Z2DBW3 | GO Process | Phosphorus metabolic process |
|  | A0A4Z2DBW3 | GO Process | Nitrogen compound metabolic process |
|  | A0A4Z2DBW3 | GO Process | Metabolic process |
|  | A0A4Z2DBW3 | GO Process | Nucleotide-sugar metabolic process |
|  | A0A4Z2DBW3 | GO Process | Cellular process |
|  | A0A4Z2DBW3 | GO Process | Energy derivation by oxidation of organic compounds |
|  | A0A4Z2DBW3 | GO Process | Cellular nitrogen compound metabolic process |
|  | A0A4Z2DBW3 | GO Process | Macromolecule metabolic process |
|  | A0A4Z2DBW3 | GO Process | Glucan metabolic process |
|  | A0A4Z2DBW3 | GO Process | Cellular metabolic process |
|  | A0A4Z2DBW3 | GO Process | Primary metabolic process |
|  | A0A4Z2DBW3 | GO Process | Cellular macromolecule metabolic process |
|  | A0A4Z2DBW3 | GO Process | Cellular carbohydrate metabolic process |
|  | A0A4Z2DBW3 | GO Process | Cellular polysaccharide metabolic process |
|  | A0A4Z2DBW3 | GO Process | Small molecule metabolic process |
|  | A0A4Z2DBW3 | GO Process | Heterocycle metabolic process |
|  | A0A4Z2DBW3 | GO Process | Nucleobase-containing small molecule metabolic process |
|  | A0A4Z2DBW3 | GO Process | Oxidation-reduction process |
|  | A0A4Z2DBW3 | GO Process | Organic substance metabolic process |
|  | A0A4Z2DBW3 | GO Process | Carbohydrate derivative metabolic process |
|  | A0A4Z2DBW3 | GO Process | Organic cyclic compound metabolic process |
|  | A0A4Z2DBW3 | GO Function | Catalytic activity |
|  | A0A4Z2DBW3 | GO Function | UTP:glucose-1-phosphate uridylyltransferase activity |
|  | A0A4Z2DBW3 | GO Function | Transferase activity |
|  | A0A4Z2DBW3 | GO Function | Transferase activity, transferring phosphorus-containing groups |
|  | A0A4Z2DBW3 | GO Function | Nucleotidyltransferase activity |
|  | A0A4Z2DBW3 | GO Function | UTP-monosaccharide-1-phosphate uridylyltransferase activity |
|  | A0A4Z2DBW3 | GO Function | Uridylyltransferase activity |
|  | A0A4Z2DBW3 | GO Component | Intracellular |
|  | A0A4Z2DBW3 | GO Component | Cytoplasm |
|  | A0A4Z2DBW3 | GO Component | Cellular anatomical entity |
|  | A0A4Z2DBW3 | KEGG | Pentose and glucuronate interconversions |
|  | A0A4Z2DBW3 | KEGG | Galactose metabolism |
|  | A0A4Z2DBW3 | KEGG | Starch and sucrose metabolism |
|  | A0A4Z2DBW3 | KEGG | Amino sugar and nucleotide sugar metabolism |
|  | A0A4Z2DBW3 | KEGG | Metabolic pathways |
| T-complex protein 1 subunit beta | A0A4Z2DPA6 | GO Process | Protein folding |
|  | A0A4Z2DPA6 | GO Process | Cellular process |
|  | A0A4Z2DPA6 | GO Function | Nucleotide binding |
|  | A0A4Z2DPA6 | GO Function | Binding |
|  | A0A4Z2DPA6 | GO Function | Protein binding |
|  | A0A4Z2DPA6 | GO Function | ATP binding |
|  | A0A4Z2DPA6 | GO Function | Purine nucleotide binding |
|  | A0A4Z2DPA6 | GO Function | Adenyl nucleotide binding |
|  | A0A4Z2DPA6 | GO Function | Ribonucleotide binding |
|  | A0A4Z2DPA6 | GO Function | Purine ribonucleotide binding |
|  | A0A4Z2DPA6 | GO Function | Adenyl ribonucleotide binding |
|  | A0A4Z2DPA6 | GO Function | Purine ribonucleoside triphosphate binding |
|  | A0A4Z2DPA6 | GO Function | Small molecule binding |
|  | A0A4Z2DPA6 | GO Function | Ion binding |
|  | A0A4Z2DPA6 | GO Function | Anion binding |
|  | A0A4Z2DPA6 | GO Function | Unfolded protein binding |
|  | A0A4Z2DPA6 | GO Function | Organic cyclic compound binding |
|  | A0A4Z2DPA6 | GO Function | Carbohydrate derivative binding |
|  | A0A4Z2DPA6 | GO Function | Nucleoside phosphate binding |
|  | A0A4Z2DPA6 | GO Function | Heterocyclic compound binding |
|  | A0A4Z2DPA6 | GO Component | Intracellular |
|  | A0A4Z2DPA6 | GO Component | Cytoplasm |
|  | A0A4Z2DPA6 | GO Component | Cytosol |
|  | A0A4Z2DPA6 | GO Component | Chaperonin-containing T-complex |
|  | A0A4Z2DPA6 | GO Component | Protein-containing complex |
|  | A0A4Z2DPA6 | GO Component | Chaperone complex |
|  | A0A4Z2DPA6 | GO Component | Cellular anatomical entity |
|  | A0A4Z2DPA6 | KEGG | Spliceosome |
| Nucleolysin TIA-1 | A0A4Z2DX64 | GO Process | Cellular process |
|  | A0A4Z2DX64 | GO Process | Regulation of gene expression |
|  | A0A4Z2DX64 | GO Process | Regulation of nucleobase-containing compound metabolic process |
|  | A0A4Z2DX64 | GO Process | Regulation of metabolic process |
|  | A0A4Z2DX64 | GO Process | Regulation of cellular metabolic process |
|  | A0A4Z2DX64 | GO Process | Regulation of biological process |
|  | A0A4Z2DX64 | GO Process | Regulation of cellular process |
|  | A0A4Z2DX64 | GO Process | Regulation of nitrogen compound metabolic process |
|  | A0A4Z2DX64 | GO Process | Regulation of RNA metabolic process |
|  | A0A4Z2DX64 | GO Process | Regulation of macromolecule metabolic process |
|  | A0A4Z2DX64 | GO Process | Biological regulation |
|  | A0A4Z2DX64 | GO Process | Regulation of primary metabolic process |
|  | A0A4Z2DX64 | GO Function | Nucleic acid binding |
|  | A0A4Z2DX64 | GO Function | RNA binding |
|  | A0A4Z2DX64 | GO Function | mRNA binding |
|  | A0A4Z2DX64 | GO Function | Binding |
|  | A0A4Z2DX64 | GO Function | Organic cyclic compound binding |
|  | A0A4Z2DX64 | GO Function | Heterocyclic compound binding |
|  | A0A4Z2DX64 | GO Component | Intracellular |
|  | A0A4Z2DX64 | GO Component | Nucleus |
|  | A0A4Z2DX64 | GO Component | Cytoplasm |
|  | A0A4Z2DX64 | GO Component | Cytoplasmic stress granule |
|  | A0A4Z2DX64 | GO Component | Protein-containing complex |
|  | A0A4Z2DX64 | GO Component | Ribonucleoprotein granule |
|  | A0A4Z2DX64 | GO Component | Cytoplasmic ribonucleoprotein granule |
|  | A0A4Z2DX64 | GO Component | Organelle |
|  | A0A4Z2DX64 | GO Component | Membrane-bounded organelle |
|  | A0A4Z2DX64 | GO Component | Non-membrane-bounded organelle |
|  | A0A4Z2DX64 | GO Component | Intracellular organelle |
|  | A0A4Z2DX64 | GO Component | Intracellular membrane-bounded organelle |
|  | A0A4Z2DX64 | GO Component | Intracellular non-membrane-bounded organelle |
|  | A0A4Z2DX64 | GO Component | Supramolecular complex |
|  | A0A4Z2DX64 | GO Component | Cellular anatomical entity |
|  | A0A4Z2DX64 | GO Component | Ribonucleoprotein complex |
|  | A0A4Z2DX64 | KEGG | Spliceosome |
| UDP-glucose 4-epimerase | C1L779 | GO Process | Carbohydrate metabolic process |
|  | C1L779 | GO Process | Monosaccharide metabolic process |
|  | C1L779 | GO Process | Galactose metabolic process |
|  | C1L779 | GO Process | Metabolic process |
|  | C1L779 | GO Process | Catabolic process |
|  | C1L779 | GO Process | Carbohydrate catabolic process |
|  | C1L779 | GO Process | Hexose metabolic process |
|  | C1L779 | GO Process | Hexose catabolic process |
|  | C1L779 | GO Process | Galactose catabolic process |
|  | C1L779 | GO Process | Galactose catabolic process via UDP-galactose |
|  | C1L779 | GO Process | Primary metabolic process |
|  | C1L779 | GO Process | Small molecule metabolic process |
|  | C1L779 | GO Process | Small molecule catabolic process |
|  | C1L779 | GO Process | Monosaccharide catabolic process |
|  | C1L779 | GO Process | Organic substance metabolic process |
|  | C1L779 | GO Process | Organic substance catabolic process |
|  | C1L779 | GO Function | Catalytic activity |
|  | C1L779 | GO Function | UDP-N-acetylglucosamine 4-epimerase activity |
|  | C1L779 | GO Function | UDP-glucose 4-epimerase activity |
|  | C1L779 | GO Function | Isomerase activity |
|  | C1L779 | GO Function | Racemase and epimerase activity |
|  | C1L779 | GO Function | Racemase and epimerase activity, acting on carbohydrates and derivatives |
|  | C1L779 | GO Component | Intracellular |
|  | C1L779 | GO Component | Cytoplasm |
|  | C1L779 | GO Component | Cytosol |
|  | C1L779 | GO Component | Cellular anatomical entity |
|  | C1L779 | KEGG | Galactose metabolism |
|  | C1L779 | KEGG | Amino sugar and nucleotide sugar metabolism |
|  | C1L779 | KEGG | Metabolic pathways |
